# A ferroelectric proton conductor with colossal polarization induced by in-plane symmetry breaking in a two-dimensional coordination polymer†

**DOI:** 10.1039/d4sc08700c

**Published:** 2025-06-26

**Authors:** Yanqing Song, Yuta Tsuji, Kunihisa Sugimoto, Takashi Kikuchi, Yuxin Shi, Yusuke Murakami, Kotaro Hiramatsu, Benjamin Le Ouay, Masaaki Ohba, Ryo Ohtani

**Affiliations:** a Department of Chemistry, Faculty of Science, Kyushu University 744 Motooka Nishi-ku Fukuoka 819-0395 Japan ohtani@chem.kyushu-univ.jp; b Faculty of Engineering Sciences, Kyushu University Kasuga Fukuoka 816-8580 Japan; c Department of Chemistry, Kindai University 3-4-1 Kowakae Higashi-osaka Osaka 577-8502 Japan; d Rigaku Corporation 3-9-12 Matsubara-cho Akishima-shi Tokyo 196-8666 Japan; e Ph.D. Program in Humanics, University of Tsukuba 1-1-1 Tennodai Tsukuba Ibaraki 305-8577 Japan

## Abstract

Noncentrosymmetric two-dimensional (2D) coordination polymers/metal–organic frameworks (CPs/MOFs) are very rare and their functionalities have not been explored. Herein, we report the first 2D ferroelectric proton conductor based on cyanido-bridged undulating 2D CPs. [Mn(salen)]_2_[ReN(CN)_4_(MeCN)]·H_2_O (MnReMeCN·H_2_O) crystallized in the *Pna*2_1_ space group and underwent in-plane symmetry breaking upon incorporation of water within the crystal layers. Thus, the dehydrated MnReMeCN layers were centrosymmetric and exhibited reversible switching of second harmonic generation induced by water vapor. The ferroelectricity of MnReMeCN·H_2_O was strongly coupled with ion conduction, yielding a colossal polarization of 21 mC cm^−2^, which was determined *via* positive-up–negative-down measurements at 0.005 Hz and 298 K for single crystals. Moreover, MnReMeCN exhibited anisotropic thermal expansion based on the undulation change, while the zigzag angle changes of the layers switched between a decrease and an increase at around 300 K. These transformations corroborate the characteristic relationship between the zigzag pitch and the interlayer interaction of the undulating layered structures.

## Abstract 2

This work reports the first 2D ferroelectric proton conductor consisting of cyanido-bridged undulating coordination layers exhibiting polarity switching and a colossal polarization of 21 mC cm^−2^ based on long-range ion displacement.

## Graphical abstract

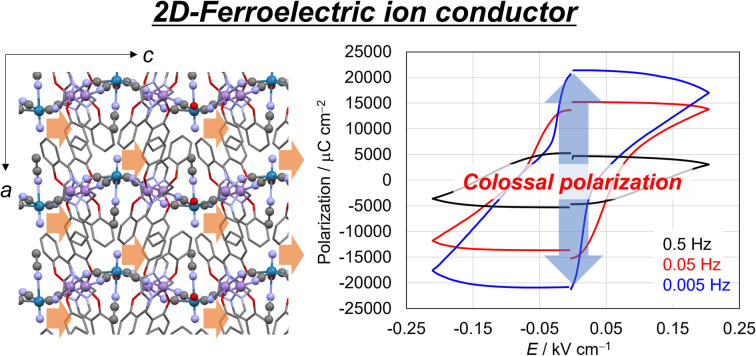

## Introduction

Two-dimensional (2D) coordination polymers/metal–organic frameworks (CPs/MOFs) are modern functional materials consisting of coordination layered structures,^1–5^ in which the anisotropic structural design of the layers allows tailoring functionalities such as magnetic,^6,7^ electronic,^8–12^ and adsorption properties.^13,14^ Among the various structural motifs of the layers, noncentrosymmetric layers constitute a fascinating structure with symmetry breaking that provides polar functionalities such as ferro-, pyro-, and piezoelectricity and second harmonic generation (SHG) activity.^15–21^ However, the design and synthesis of such noncentrosymmetric structures is difficult, which renders the synthesis of noncentrosymmetric 2D CPs/MOFs and the investigation of their ferroelectricity challenging tasks.

Recently, multifunctionalities based on polarity have been investigated by combining magnetism, luminescence, ion conduction, and guest responsivity.^22–29^ Metal-complex-based solid-state materials are composed of characteristic flexible frameworks with coordination networks, yielding host–guest polar systems. Notably, the incorporation of guest species such as water within the lattice of polarity-switchable crystals has been demonstrated to induce symmetry breaking.^28–31^ In addition, the presence of guest water in polar crystals leads to the formation of specific hydrogen bonding, which results in unique functionalities such as super-ion conduction^32^ and ferroelectricity.^33,34^ Importantly, recent experimental and theoretical investigations of such ion-conductive polar systems suggested that long-range ion displacement in crystals causes anomalous polarization phenomena, giving rise to ferroelectric ion conductors.^35,36^ This long-range ion displacement constitutes a new polarization mechanism involving strong correlation between the conduction ions and the polar skeleton. Compared with conventional displacement-type and order–disorder-type transitions, a large polarization greater than 1 mC cm^−2^ is achieved. Thus, exploring new systems underpinned by long-range ion displacement is important for the development of advanced ferroelectric materials. However, coupling ferroelectricity with ion conduction in polar crystals is challenging and still rare.^33,34^

**Fig. 1:**
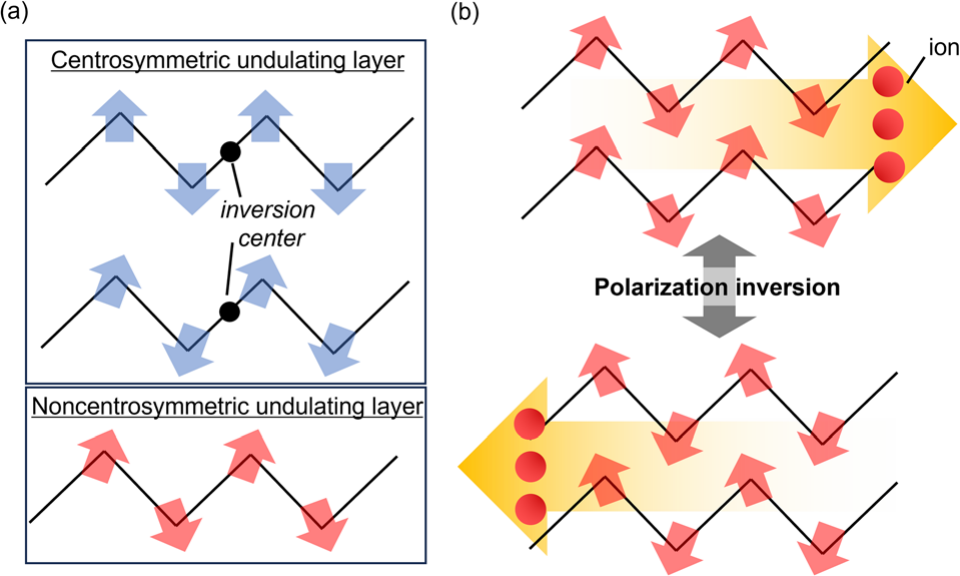
(a) Structural design of noncentrosymmetric layers based on undulation. (b) Schematic of the polarization inversion of a 2D ferroelectric ion conductor based on long-range ion displacement.

Our group investigated undulating layer–type 2D CPs/MOFs, in particular [M(salen)]_2_[M′(CN)_4_(solvent)] (M = Mn and Fe; M′ = MnN, ReN, Pt, and PtI_2_; solvent = MeOH and MeCN), and explored their anisotropic properties, such as thermal expansion, compression, and ion conduction.^37–39^ Unlike flat layers, undulating layers contain tunable alignments of dipoles *via* in-plane distortion (Fig. 1a). Notably, the [ReN(CN)_4_]^2−^ unit, which has been extensively studied as a luminescent complex,^40–44^ was found to be a characteristic building block to construct undulating layers owing to its unique umbrella-shaped geometry with a dipole. For example, [Mn^III^(salen)]_2_[Re^V^(CN)_4_(H_2_O)_2_]·H_2_O incorporated in-plane distortion due to interlayer interaction, resulting in dipole cancellation; therefore, centrosymmetric crystals were obtained.^45^ This result indicated that effectively controlling the direction of in-plane distortion within undulating 2D CP structures could lead to polar structures exhibiting ion conduction. On the basis of this design idea, we further explored the polar structures and their functionalities *via* layer modification using other solvent molecules (Fig. 1a).

In this study, [Mn^III^(salen)]_2_[Re^V^N(CN)_4_(MeCN)]·H_2_O (MnReMeCN·H_2_O) was synthesized as the first 2D ferroelectric proton conductor. This compound exhibited water-dependent SHG-switching *via* in-plane symmetry breaking of layers and ferroelectricity with an extremely large polarization (21 mC cm^−2^, 0.005 Hz) coupled with ion transport (Fig. 1b). Moreover, dehydrated nonpolar MnReMeCN showed switching of the anisotropic thermal expansion (ATE) with undulation changes at 300 K, resulting in a characteristic relationship between the layer undulation and interlayer distances.

## Results and discussion

### Crystal structures and polarity switching

**Fig. 2:**
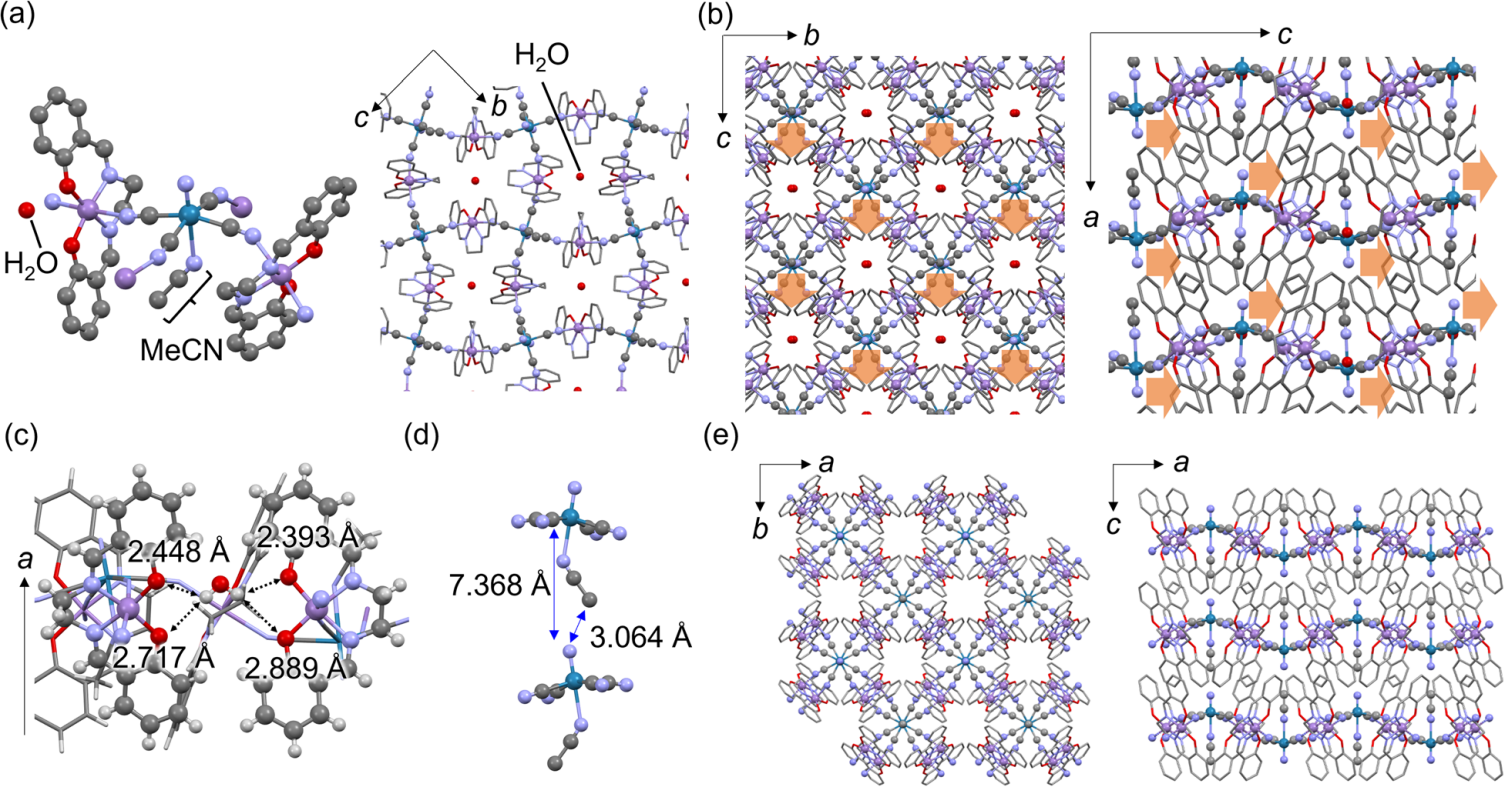
(a) Crystal structures and (b) packing views of MnReMeCN·H_2_O. Orange arrows indicate the displacement of nitrido groups in the *c*-axis direction causing polarization. Color code: purple (Mn), green (Re), red (O), blue (N), and gray (C). Hydrogen atoms are omitted for clarity. (c) Hydrogen bonding between lattice water and the oxygen atoms of the surrounding salen ligands. (d) Interlayer distance, *i.e.*, the distance between Re and the nitrido group of the adjacent layer (7.368 Å) and distance between the nitrido group and the closest MeCN carbon (3.064 Å). (e) Crystal structure of MnReMeCN.

Block-type single crystals of MnReMeCN·H_2_O were synthesized by slowly mixing MeCN solutions of (PPh_4_)_2_[ReN(CN)_4_(MeOH)]·3MeOH^44^ and water solutions of [Mn(salen)(H_2_O)_2_]Cl (Fig. S1†). At 100 K, MnReMeCN·H_2_O crystallized in the orthorhombic noncentrosymmetric *Pna*2_1_ space group (Table S1†). MnReMeCN·H_2_O consists of cyanido-bridged undulating layers expanding in the *bc* plane and stacking in the *a* axis direction (Fig. 2a and b). MeCN coordinates to the axial position of the [ReN(CN)_4_]^2−^ units. Water exists at the center of the grids and forms hydrogen bonds with the oxygen atoms of the salen ligands (Fig. 2c). This water is removed at 350 K, as confirmed by TG-DTA (Fig. S2†). Uniquely, the symmetry breaking of the layered structures of MnReMeCN·H_2_O involves in-plane distortion caused by the interlayer interaction between nitrido groups and MeCN moieties (Fig. 2d). Due to their repulsion, the Re≡N group is tilted at 4.3° along the *c*-axis direction and MeCN is tilted from the *a* axis. The polarization value of MnReMeCN·H_2_O was calculated to be 0.57 μC cm^−2^ on the basis of its crystal structure (Fig. S3†).

The lattice water within the grid was found to be responsible for the symmetry breaking of the layers. Dehydrated MnReMeCN crystallized in the *P*4/*ncc* space group at 400 K (Fig. 2e and Table S2†). The cyanido-bridged layers remained intact, but the in-plane distortion disappeared with the removal of the lattice water. This demonstrates that MnReMeCN is a polarity-switchable 2D CP based on displacive-type polarization in response to water adsorption. MnReMeCN gradually adsorbed water molecules with a hysteresis at room temperature, indicating a slow response to water vapor likely due to its dense structure (Fig. S4†). The cyclability of the reversible polarity switching associated with structural transformations induced by water adsorption was confirmed *via* SHG, PXRD and water adsorption measurements (Fig. S4–S6†).

A comparison between MnReMeCN and its [Mn(salen)]_2_[MnN(CN)_4_(MeCN)] (MnMnMeCN)^46^ and [Fe(salen)]_2_[MnN(CN)_4_(MeCN)] (FeMnMeCN)^37^ analogs suggested that the water responsivity associated with the structural transformation of the undulating layers affects the relationship between the undulating layer size and the flexibility. In particular, MnReMeCN exhibited the largest layers, reflecting its large metal-ion size (Mn^III^ > Fe^III^ and Re^V^ > Mn^V^; Fig. S7†). The medium-sized grids of MnMnMeCN accommodate water molecules, resulting in the conversion of the space group from *P*4/*ncc* to *Pccn* without tilting MeCN,^46^ whereas the smallest grids of FeMnMeCN provide no space for accommodating water molecules.^37^ Meanwhile, [Mn(salen)]_2_[ReN(CN)_4_(H_2_O)]·2H_2_O exhibits a similar in-plane distortion but crystallizes in the nonpolar *Pbcn* space group.^45^ These results indicate that the rigidity of MeCN within the large layers accounts for the resultant in-plane symmetry breaking of the coordination layers.

### Ferroelectricity coupled with ion conduction

**Fig. 3:**
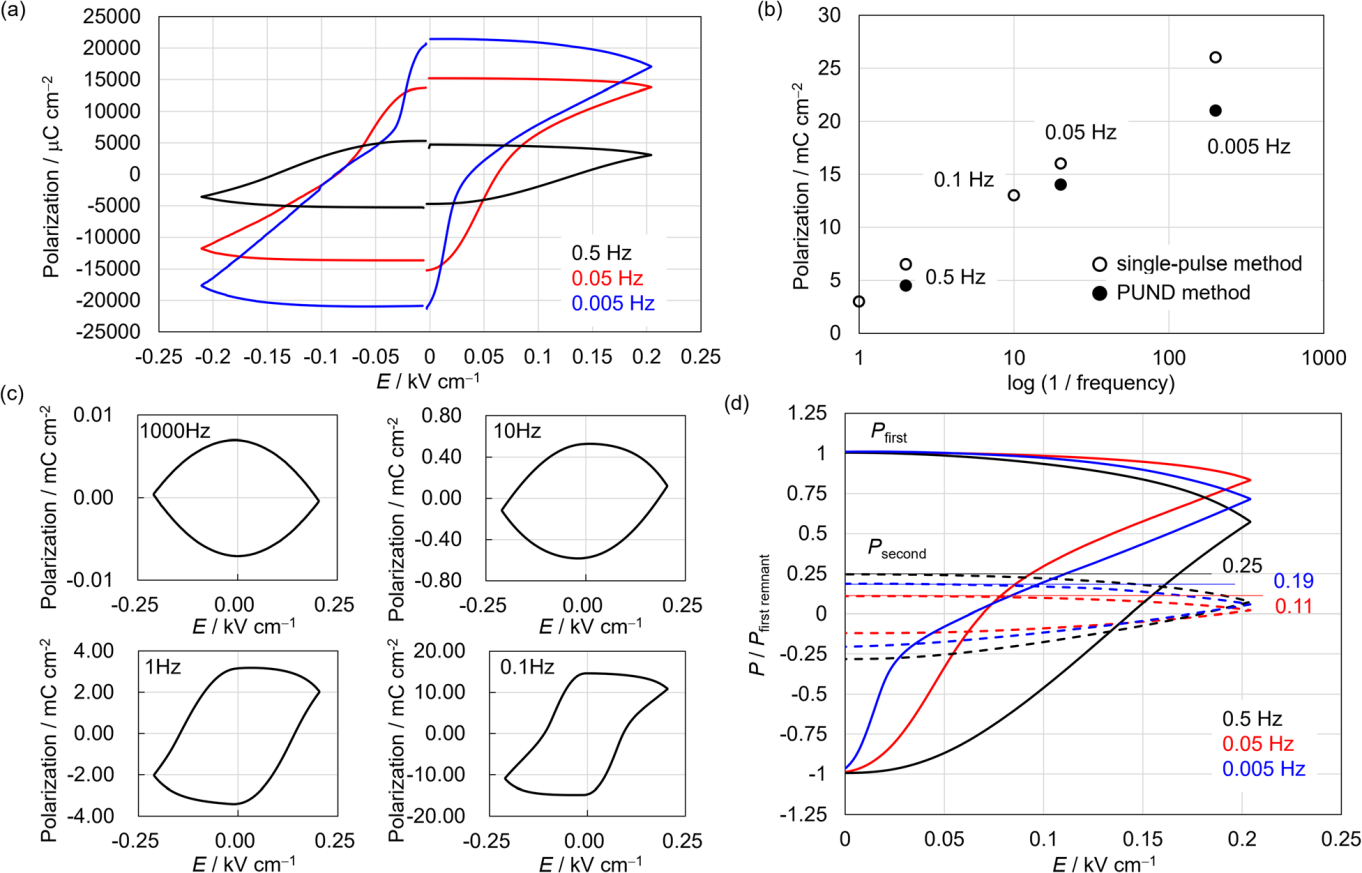
(a) Polarization–electric field hysteresis loops obtained *via* PUND measurements. (b) Polarization as a function of frequency. (c) Frequency dependency on the hysteresis loops. (d) Frequency dependency on the polarization behavior of the first and second pulses. The vertical axis is the ratio of the polarization of the first or second pulse and the remnant polarization value of the first pulse. *P*_first or second_ = *I*_first or second pulse_ × *t*.

**Fig. 4:**
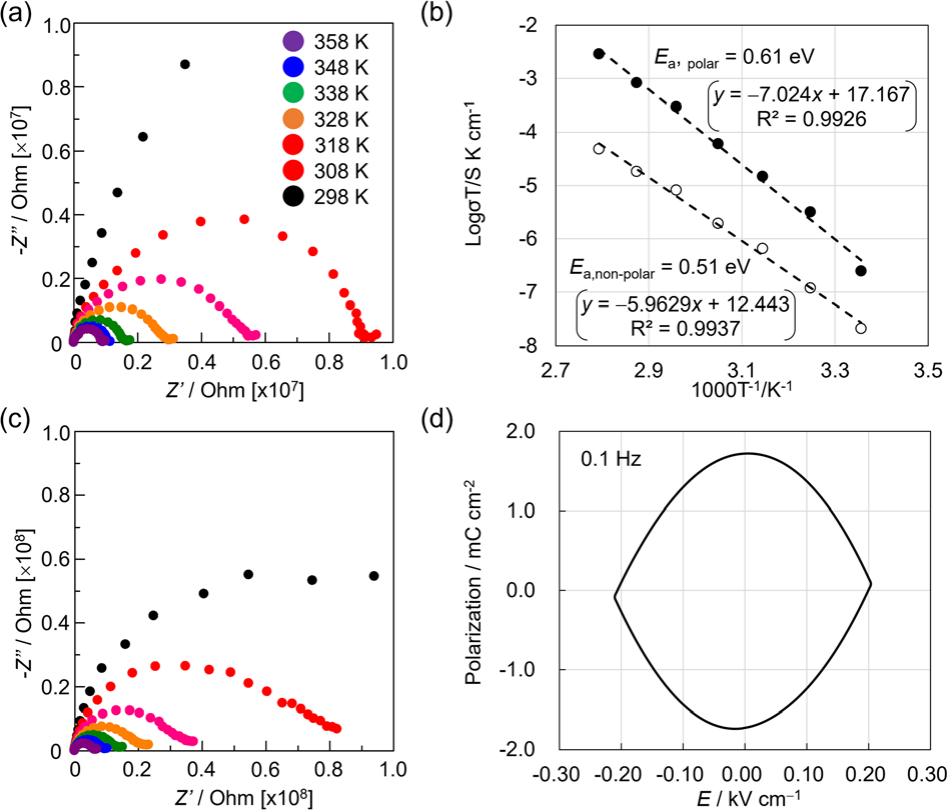
(a) Variable-temperature Nyquist plots of a MnReMeCN·H_2_O single crystal in the [001] direction at 85% relative humidity. (b) Arrhenius plots of MnReMeCN·H_2_O in the [001] (closed circles) and [110] or [1−10] (open circles) directions. (c) Variable-temperature Nyquist plots of a MnReMeCN·H_2_O single crystal in the [110] or [1−10] direction at 85% relative humidity. (d) Polarization–electric field measurement result for the nonpolar direction of MnReMeCN·H_2_O.

Noncentrosymmetric MnReMeCN·H_2_O exhibited a notable ferroelectricity with a colossal remnant polarization of 21 mC cm^−2^ at room temperature and a relative humidity of 85%, as revealed by a positive-up–negative-down (PUND) measurement performed at 0.005 Hz on single crystals (Fig. 3). The voltage for the polarization inversion was 0.04 kV cm^−1^. Uniquely, as the frequency became faster, the polarization inversion voltage became higher, whereas the polarization value decreased considerably (Fig. 3a and b and S8†). Moreover, no hysteresis was observed at 10–1000 Hz (Fig. 3c). In addition to the characteristic frequency-dependent hysteretic behavior, the proton conduction of MnReMeCN·H_2_O was confirmed by conducting variable-temperature AC impedance measurements on a crystal, which yielded a conductivity of 3.53 × 10^−6^ S cm^−1^ at 298 K and an activation energy (*E*_a_) of 0.61 eV (Fig. 4a and b). By studying the isotope effect using D_2_O on conductivity (Fig. S9†), we confirmed that proton conduction was caused by proton hopping *via* water molecules in the lattice.^47,48^ Note that the water–layer interaction *via* hydrogen bonds facilitates efficient proton dissociation for conduction. The cyanido and nitrido groups of the layers would connect the pathways through which protons flow. These results demonstrate that MnReMeCN·H_2_O is a ferroelectric proton conductor^33^ based on long-range ion displacement, whose ferroelectricity is strongly correlated with proton conduction (Fig. 1b). The proton flow within the lattice amplifies the current involved in the ferroelectric response; that is, the number of conducting and trapped protons increases at slower frequencies, resulting in an increase in the polarization value of the ferroelectricity (Fig. 3b and S10†). As a consequence, the polarization value of MnReMeCN·H_2_O is much higher than that calculated using the crystal structure (0.57 μC cm^−2^). Moreover, the decrease in the voltage of polarization inversion at slower frequency is in accord with the protons reaching the electrode interface (Fig. S8†), which strongly supports the proton conduction behavior.

Ferroelectric measurements conducted on several single crystals of MnReMeCN·H_2_O revealed that several crystals exhibited no hysteresis but IV curves based on ion conduction (Fig. 4d). This result is in accordance with the results observed for the [110] and [1−10] directions of the selected crystals. The proton conductivity in the nonpolar direction was 1.55 × 10^−6^ S cm^−1^ at 298 K and *E*_a_ = 0.51 eV (Fig. 4b and c). The crystal morphology changed considerably after high-voltage (100 V) measurements along the [001] direction (Fig. S11†). Such macroscopic changes are most likely associated with the electric-field-induced structural transformation leading to polarization inversion, indicating the weakness of the 2D crystal formed *via* van der Waals interactions between the layers. This hypothesis is supported by the fact that no such morphological changes were observed when the P–E measurements were performed along the nonpolar axis (*i.e.,* the [110] or [1–10] direction).

To gain insight into the synergistic properties of polarity inversion and proton flow, the frequency dependency on the current was investigated using the first and second pulses in PUND measurements, demonstrating that the matching dynamics of the skeletons and conduction ions responding to the electric fields are responsible for the anomalous polarization. Specifically, the ratio of the calculated polarization value using the second pulse (*P*_second_ = *I*_second pulse_ × *t*) and the remnant polarization value of the first pulse (*P*_first remnant_) changed nonlinearly (Fig. 3d). *P*_second_/*P*_first remnant_ showed the lowest value of 0.11 at 0.05 Hz and values of 0.25 and 0.19 at 0.5 and 0.005 Hz, respectively. *I*_second pulse_ is caused by the conduction of free ions within the channel; that is, when the frequency exceeds a certain threshold, the *P*_second_/*P*_first remnant_ increases as more untrapped ions return from the electrode interface where the ions are biased. Thus, the maximum amount of ions involved in the polarization of the ferroelectric ion conductor is determined by the characteristic interaction between the polar skeletons and the conduction ions.

Although a similar proton-conduction-coupled ferroelectricity was demonstrated for K_2_MnN(CN)_4_·H_2_O,^33^ the voltage for the polarization inversion of MnReMeCN·H_2_O (0.09 kV cm^−1^ at 0.1 Hz) was considerably lower than that of K_2_MnN(CN)_4_·H_2_O (0.5 kV cm^−1^ at 0.1 Hz). This decrease in voltage indicates the occurrence of different polarization mechanisms in MnReMeCN·H_2_O and K_2_MnN(CN)_4_·H_2_O. In K_2_MnN(CN)_4_·H_2_O, polarization proceeds through a combination of order–disorder and displacive mechanisms with nitrido migration *via* bond cleavage and reformation. Conversely, MnReMeCN·H_2_O only displays displacive-type polarization, which requires a change in the layer distortion direction for the polarization inversion, resulting in a low ferroelectric voltage. In addition, the single-pulse and PUND measurements gave relatively close polarization values of 26 and 21 mC cm^−2^, respectively, for MnReMeCN·H_2_O. In contrast, for K_2_MnN(CN)_4_·H_2_O, the polarization obtained *via* the PUND method (56 mC cm^−2^) was half of that obtained *via* the single-pulse method (120 mC cm^−2^). These facts suggest that compared with K_2_MnN(CN)_4_·H_2_O, MnReMeCN·H_2_O incorporates fewer untrapped protons in the polar skeleton and its leakage current is smaller. The leakage current in ferroelectric behavior with long-range ion displacement is associated with the irreversibility of proton migration. Thus, the *E*_a_ of proton conduction would be significant for such characteristic ferroelectric responses. MnReMeCN·H_2_O and K_2_MnN(CN)_4_·H_2_O exhibited noticeably different *E*_a_ values owing to their proton path structures, *i.e.*, hydrophobic interlayer narrow spaces (*E*_a_ = 0.61 eV) and hydrophilic 1D channels surrounded with cyanido groups (*E*_a_ = 0.48 eV), respectively. This suggests that a high *E*_a_ could be beneficial for efficiently using the conduction ions for polarization, although it is not favorable for proton conduction.

### Water diffusion behavior

**Fig. 5:**
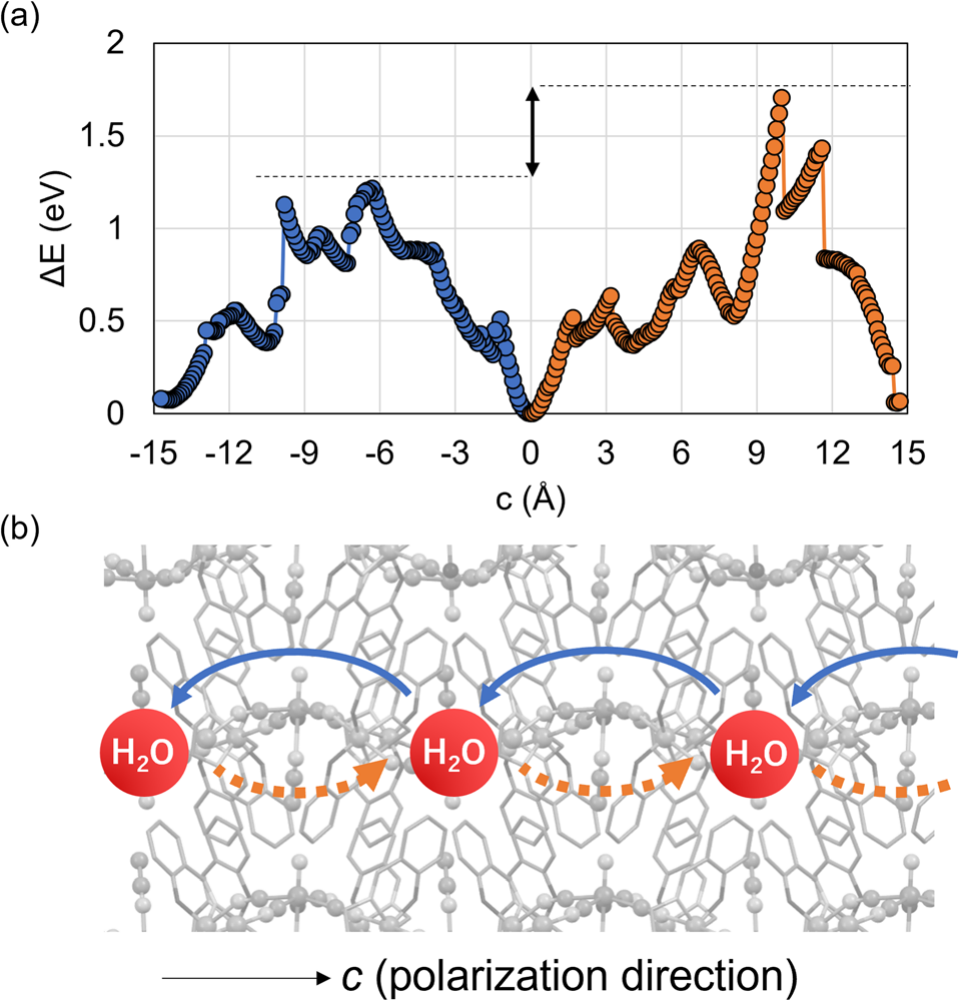
(a) Changes in the potential energy calculated for MnReMeCN·H_2_O when water is displaced in the +*c* (orange) and −*c* (blue) directions. (b) Schematic of the water migration paths in the +*c* and −*c* directions.

Guest diffusion in crystals is an important phenomenon involved in various functions, such as adsorption, separation, guest-induced structural changes, and ion conduction.^49–52^ In this sense, it is important to clarify the effect of noncentrosymmetric structures on such diffusion behavior. Thus, to obtain insight into the water migration in the MnReMeCN·H_2_O framework, the energy differences associated with water flow were calculated by changing the water position between the +*c*- and −*c*-axis directions and between the *a* and *b* axes (Fig. 5 and S12†). It was found that water flows more easily in the nonpolar *a*- and *b*-axis directions than in the *c*-axis direction. Importantly, the calculated *E*_a_ values along the *c*-axis are different in the +*c-* and −*c*-axis directions, with the *E*_a_ in the −*c* direction being lower than that in the +*c* direction. This directional migration behavior occurs due to the existence of different migration paths stemming from the noncentrosymmetric layered structure with the tilted N≡Re–MeCN moieties; specifically, water molecules pass through the Re≡N side in the +*c* axis and through the MeCN side in the −*c*-axis direction (Fig. 5b and S13†). Note that directional migration is related to rectification, which has been widely explored in various fields for the construction of sophisticated functional devices that actively transport ions and molecules.^53–55^ In this context, we recently reported that the rectified water migration behavior of K_2_MnN(CN)_4_·H_2_O also occurred due to the different molecular motions of water depending on the direction along the noncentrosymmetric 1D channel.^56^ These structure–guest migration relationships prove that polar structures cause unique molecular dynamics and intracrystalline directional diffusion behaviors.

### Anisotropic thermal expansion (ATE)

**Fig. 6:**
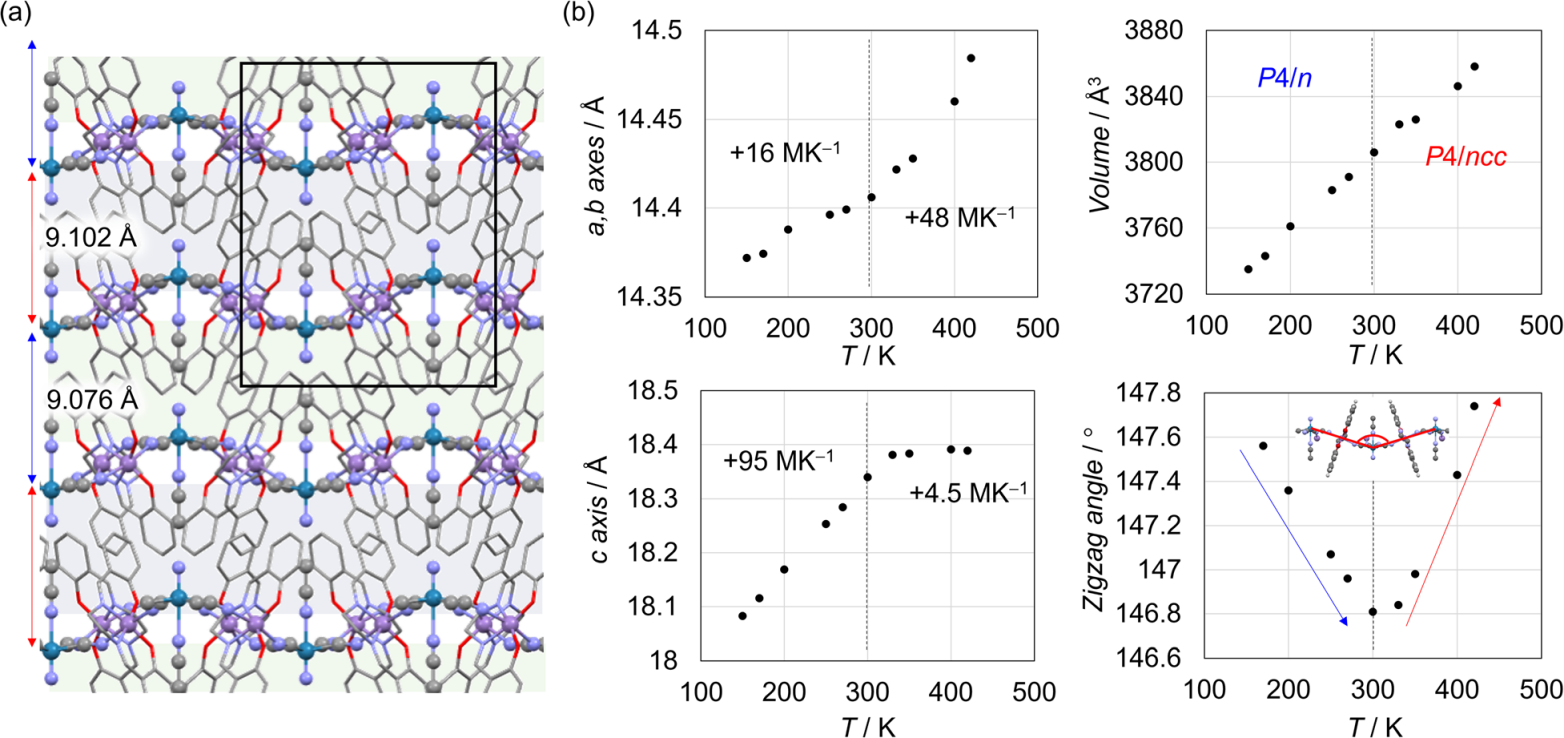
(a) Crystal structure of MnReMeCN_LT_ (*P*4/*n*) at 200 K. The black square indicates the unit cell. (b) Results of the thermal expansion behavior of MnReMeCN in terms of the thermal variation of the cell parameters, cell volume, and angles between the tetracyanometallate units in the zigzag layer.

The strong interlayer interaction in the MnReMeCN framework was found to affect considerably the thermally induced changes of the undulating layered structures associated with ATE. The thermal expansion of coordination frameworks has been actively investigated because of their structural flexibility based on dynamic connections and structural anisotropy.^57–65^ In our previous investigation of the ATE of a series of [M(salen)]_2_[M′(CN)_4_(solvent)] compounds (M = Mn and Fe; M′ = MnN, ReN, Pt and PtI_2_; solvent = MeOH and MeCN), we demonstrated how undulation changes and interlayer interactions influence the ATE behavior.^37^ In the present study, variable-temperature SC-XRD revealed an anomaly in the ATE of MnReMeCN at around 300 K (Fig. 6 and Table 1). More specifically, the space group changed between *P*4/*ncc* at higher temperatures (HT) and *P*4/*n* at lower temperatures (LT) (Fig. 6a and Table S2†). Accordingly, although the volume changes were nearly linear at 150–420 K (*α*_*V*_ = d*V*/*V*d*T* = 100–103 MK^−1^; *V* = crystal volume, *T* = temperature, *M* = 10^−6^), this phase transition at 300 K largely affected the ATE behavior linked with the structural changes of the undulating layers. Structural analyses revealed the unique tendency of the undulation change at 300 K; that is, the undulation angle of the layers decreased in the LT region and oppositely increased in the HT region with increasing temperature (Fig. 6b). Since the structural transformations around the [Mn(salen)]^+^ moieties were similar and the layer area expanded at high and low temperatures, the interlayer repulsion between the nitrido and MeCN moieties due to the narrow layer stacking of MnReMeCN accounts for this unique anisotropy switch during the thermal expansion (TE) behavior (Fig. S14†). Specifically, MnReMeCN_LT_, showing a short stacking distance with strong interlayer repulsion, exhibited a high *α*_*c*_ (d*l*_*c*_/*l*_*c*_d*T* = +100 M K^−1^; *l*_*c*_ = *c*-axis value) and a displacement of the [ReN(CN)_4_(MeCN)]^2−^ units along the *c*-axis direction to decrease the zigzag angles of the layers. This structural transformation largely differs from that observed for [Mn(salen)]_2_[MN(CN)_4_] analogs (M = Mn and Re), which exhibit similar decreases in the zigzag angles but a negative area TE.^45^ Meanwhile, in the HT region where the interlayer repulsion is weak, the large area TE (*α*_*a*_ = d*l*_*a*_/*l*_*a*_d*T* = +48 MK^−1^; *l*_*a*_ = *a* axis value) of MnReMeCN_HT_ caused a large decrease in the layer thickness, yielding a nearly zero *α*_*c*_ (+4.5 MK^−1^) by counterbalancing the interlayer space expansion. Note that this low *α*_*c*_ of MnReMeCN_HT_ was also observed for an analog of MnMnMeCN (Table 1).^37,46^

**Table 1:** Thermal expansion coefficients for isostructural compounds of MM′MeCN (M = Mn and Fe; M′ = Re and Mn)

	Space group	*α*_*a*_ (MK^−1^)	*α*_*c*_ (MK^−1^)	*α*_*V*_ (MK^−1^)	Ref.
MnReMeCN_HT_ (300–420 K)	*P*4/*ncc*	+48	+4.5	+100	This work
MnReMeCN_LT_ (150–270 K)	*P*4/*n*	+16	+95	+103	This work
MnMnMeCN (150–400 K)	*P*4/*ncc*	+63 (29)	−6.2(31)	+120(88)	37
FeMnMeCN (150–400 K)	*P*4/*ncc*	+33(5)	+46(5)	+111(14)	37

## Conclusions

This work reports the first 2D ferroelectric proton conductor consisting of cyanido-bridged undulating coordination layers exhibiting polarity switching and a colossal polarization of 21 mC cm^−2^ based on long-range ion displacement. The undulating layers with in-plane distortion are a unique structural motif capable of inducing polarization inversion. Such a unique property is not observed in Janus-type layered structures, which are other noncentrosymmetric motifs in 2D materials.^66^ Thus, understanding and controlling distortion within the undulating layers allows modulating the characteristic symmetry breaking of the layers for the construction of novel functional polar materials. This work also illustrates that 2D materials offer a powerful platform for the development of future ferroelectric ion conductors by coupling anisotropic ion conduction with polarity because layered structures enable the design of anisotropic functionalities based on the in-plane and out-of-plane directions.

## Author contributions

R. O. conceived and designed the project. Y. Song, Y. Shi, R. O. and B. L. O. performed all synthetic and characterization experiments. K. S. performed synchrotron radiation measurements for MnReMeCN·H_2_O. R. O. and T. K. analysed crystal structures. Y. M. and K. H. performed SHG measurements. R. O. and M. O. supervised the project. Y. Song and R. O. wrote the manuscript. All authors discussed the results and commented on the manuscript.

## Conflicts of interest

There are no conflicts to declare.

## Supplementary Material

SC-OLF-D4SC08700C-s001

SC-OLF-D4SC08700C-s002

## Data Availability

The data supporting this article have been included as part of the ESI.† Crystallographic data for MnReMeCN·H_2_O (100 K) and MnReMeCN (150, 170, 200, 250, 270, 300, 330, 350, 400 and 420 K) have been deposited at the CCDC under 2410485–2410495 and can be obtained from http://www.ccdc.cam.ac.uk/data_request/cif.

## References

[cit1] Chakraborty G., Park I. H., Medishetty R., Vittal J. J. (2021). Two-Dimensional Metal-Organic Framework Materials: Synthesis, Structures, Properties and Applications. Chem. Rev..

[cit2] Zheng Y. S., Sun F. Z., Han X., Xu J. L., Bu X. H. (2020). Recent Progress in 2D Metal-Organic Frameworks for Optical Applications. Adv. Opt. Mater..

[cit3] Nicks J., Sasitharan K., Prasad R. R. R., Ashworth D. J., Foster J. A. (2021). Metal-Organic Framework Nanosheets: Programmable 2D Materials for Catalysis, Sensing, Electronics, and Separation Applications. Adv. Funct. Mater..

[cit4] Dhakshinamoorth A., Asiri A. M., Garcia H. (2019). 2D Metal-Organic Frameworks as Multifunctional Materials in Heterogeneous Catalysis and Electro/Photocatalysis. Adv. Mater..

[cit5] Peng Y., Li Y. S., Ban Y. J., Jin H., Jiao W. M., Liu X. L., Yang W. S. (2014). Metal-organic framework nanosheets as building blocks for molecular sieving membranes. Science.

[cit6] Li J., Wu R. Q. (2020). Metal-organic frameworks: possible new two-dimensional magnetic and topological materials. Nanoscale.

[cit7] Perlepe P., Oyarzabal I., Mailman A., Yquel M., Platunov M., Dovgaliuk I., Rouzieres M., Negrier P., Mondieig D., Suturina E. A., Dourges M. A., Bonhommeau S., Musgrave R. A., Pedersen K. S., Chernyshov D., Wilhelm F., Rogalev A., Mathoniere C., Clerac R. (2020). Metal-organic magnets with large coercivity and ordering temperatures up to 242 degrees C. Science.

[cit8] Ko M., Mendecki L., Mirica K. A. (2018). Conductive two-dimensional metal-organic frameworks as multifunctional materials. Chem. Commun..

[cit9] Park C., Baek J. W., Shin E., Kim I. (2023). Two-Dimensional Electrically Conductive Metal-Organic Frameworks as Chemiresistive Sensors. ACS Nanosci. Au.

[cit10] Apostol P., Gali S. M., Su A., Tie D., Zhang Y., Pal S., Lin X. D., Bakuru V. R., Rambabu D., Beljonne D., Dinca M., Vlad A. (2023). Controlling Charge Transport in 2D Conductive MOFs-The Role of Nitrogen-Rich Ligands and Chemical Functionality. J. Am. Chem. Soc..

[cit11] Lu Y., Zhang Y. Y., Yang C. Y., Revuelta S., Qi H. Y., Huang C. H., Jin W. L., Li Z. C., Vega-Mayoral V., Liu Y. N., Huang X., Pohl D., Polozij M., Zhou S. Q., Cánovas E., Heine T., Fabiano S., Feng X. L., Dong R. H. (2022). Precise tuning of interlayer electronic coupling in layered conductive metal-organic frameworks. Nat. Commun..

[cit12] Kambe T., Sakamoto R., Hoshiko K., Takada K., Miyachi M., Ryu J. H., Sasaki S., Kim J., Nakazato K., Takata M., Nishihara H. (2013). π-Conjugated Nickel Bis(dithiolene) Complex Nanosheet. J. Am. Chem. Soc..

[cit13] Sakaida S., Otsubo K., Sakata O., Song C., Fujiwara A., Takata M., Kitagawa H. (2016). Crystalline coordination framework endowed with dynamic gate-opening behaviour by being downsized to a thin film. Nat. Chem..

[cit14] Tanaka D., Henke A., Albrecht K., Moeller M., Nakagawa K., Kitagawa S., Groll J. (2010). Rapid preparation of flexible porous coordination polymer nanocrystals with accelerated guest adsorption kinetics. Nat. Chem..

[cit15] Runowski M., Marcinkowski D., Soler-Carracedo K., Gorczynski A., Ewert E., Wozny P., Martín I. R. (2023). Noncentrosymmetric Lanthanide-Based MOF Materials Exhibiting Strong SHG Activity and NIR Luminescence of Er: Application in Nonlinear Optical Thermometry. ACS Appl. Mater. Interfaces.

[cit16] Taniguchi K., Nishio M., Abe N., Huang P. J., Kimura S., Arima T. H., Miyasaka H. (2021). Magneto-Electric Directional Anisotropy in Polar Soft Ferromagnets of Two-Dimensional Organic-Inorganic Hybrid Perovskites. Angew. Chem., Int. Ed..

[cit17] Lee Y., Park J., Cho S., Shin Y. E., Lee H., Kim J., Myoung J., Cho S., Kang S., Baig C., Ko H. (2018). Flexible Ferroelectric Sensors with Ultrahigh Pressure Sensitivity and Linear Response over Exceptionally Broad Pressure Range. ASC Nano..

[cit18] Nguyen S. D., Yeon J., Kim S. H., Halasyamani P. S. (2011). BiO(IO3): A New Polar Iodate that Exhibits an Aurivillius-Type (Bi2O2)(2+) Layer and a Large SHG Response. J. Am. Chem. Soc..

[cit19] Sekine Y., Akiyoshi R., Hayami S. (2022). Recent advances in ferroelectric metal complexes. Coord. Chem. Rev..

[cit20] Shi P. P., Tang Y. Y., Li P. F., Liao W. Q., Wang Z. X., Ye Q., Xiong R. G. (2016). Symmetry breaking in molecular ferroelectrics. Chem. Soc. Rev..

[cit21] Zhang W., Xiong R. G. (2012). Ferroelectric Metal-Organic Frameworks. Chem. Rev..

[cit22] Jornet-Mollá V., Duan Y., Giménez-Saiz C., Tang Y. Y., Li P. F., Romero F. M., Xiong R. G. (2017). A Ferroelectric Iron(ii) Spin Crossover Material. Angew. Chem., Int. Ed..

[cit23] Yuan G., Kimura Y., Kobayashi T., Takeda T., Hoshino N., Akutagawa T. (2021). Ion polarisation-assisted hydrogen-bonded ferroelectrics in liquid crystalline domains. Chem. Sci..

[cit24] Zhu S. D., Hu J. J., Dong L., Wen H. R., Liu S. J., Lu Y. B., Liu C. M. (2020). Multifunctional Zn(ii)-Yb(iii) complex enantiomers showing second-harmonic generation, near-infrared luminescence, single-molecule magnet behaviour and proton conduction. J. Mater. Chem. C.

[cit25] Li M., Pietrowski M. J., De Souza R. A., Zhang H. R., Reaney I. M., Cook S. N., Kilner J. A., Sinclair D. C. (2014). A family of oxide ion conductors based on the ferroelectric perovskite Na0.5Bi0.5TiO_3_. Nat. Mater..

[cit26] Xu W. J., Li P. F., Tang Y. Y., Zhang W. X., Xiong R. G., Chen X. M. (2017). A Molecular Perovskite with Switchable Coordination Bonds for High-Temperature Multiaxial Ferroelectrics. J. Am. Chem. Soc..

[cit27] Kimura T., Goto T., Shintani H., Ishizaka K., Arima T., Tokura Y. (2003). Magnetic control of ferroelectric polarization. Nature.

[cit28] Kobayashi F., Gemba M., Hoshino S., Tsukiyama K., Shiotsuka M., Nakajima T., Tadokoro M. (2023). Polarity and Dielectric Property Control Triggered by a Coordinated Solvent Molecule Exchange in Luminescent Mononuclear Aluminium(iii) Complexes. Chem.–Eur. J..

[cit29] Kobayashi F., Akiyoshi R., Kosumi D., Nakamura M., Lindoy L. F., Hayami S. (2020). Solvent vapor-induced polarity and ferroelectricity switching. Chem. Commun..

[cit30] Yanagisawa J., Tanaka K., Kano H., Miyata K., Le Ouay B., Ohtani R., Ohba M. (2022). Vapor-Induced Conversion of a Centrosymmetric Organic-Inorganic Hybrid Crystal into a Proton-Conducting Second-Harmonic-Generation-Active Material. Inorg. Chem..

[cit31] Cui H. B., Wang Z. M., Takahashi K., Okano Y., Kobayashi H., Kobayashi A. (2006). Ferroelectric porous molecular crystal, [Mn(HCOO)](CHOH), exhibiting ferrimagnetic transition. J. Am. Chem. Soc..

[cit32] Ohkoshi S., Nakagawa K., Imoto K., Tokoro H., Shibata Y., Okamoto K., Miyamoto Y., Komine M., Yoshikiyo M., Namai A. (2020). A photoswitchable polar crystal that exhibits superionic conduction. Nat. Chem..

[cit33] Yanagisawa J., Aoyama T., Fujii K., Yashima M., Inaguma Y., Kuwabara A., Shitara K., Le Ouay B., Hayami S., Ohba M., Ohtani R. (2024). Strongly Enhanced Polarization in a Ferroelectric Crystal by Conduction-Proton Flow. J. Am. Chem. Soc..

[cit34] Lu J. L., Luo R., Zhou J. Y., Hao M. N., Chai C. C., Ying T. P., Gao Y. R., Jin S. F., Chen X. L. (2024). High, Multiple, and Nonvolatile Polarizations in Organic-Inorganic Hybrid [(CH)(CHCHCl)N]InCl·H2O for Memcapacitor. J. Am. Chem. Soc..

[cit35] Wang X. C., Ren Y. Y., Wu M. H. (2022). Unconventional Ferroelectricity with Quantized Polarizations in Ionic Conductors: High-Throughput Screening. J. Phys. Chem. Lett..

[cit36] Sheng Y. X., Wu M. H., Liu J. M. (2024). Ferroelectricity with Long ion Displacements in Crystals of Non-Polar Point Groups. Adv. Funct. Mater..

[cit37] Ohtani R., Yanagisawa J., Iwai Y., Le Ouay B., Ohba M. (2022). Negative Thermal Expansion of Undulating Coordination Layers through Interlayer Interaction. Inorg. Chem..

[cit38] Iwai Y., Kusumoto S., Suzuki R., Tachibana M., Komatsu K., Kikuchi T., Kawaguchi S. I., Kadobayashi H., Masubuchi Y., Yamamoto Y., Ozawa Y., Abe M., Hirai K., Le Ouay B., Ohba M., Ohtani R. (2024). Mechanical Properties of Modulative Undulating Layers in Two-Dimensional Metal-Organic Frameworks. Chem. Mater..

[cit39] Shi Y., Kimura S., Iwai Y., Tsuji Y., Ouay B. L., Ohba M., Ohtani R. (2024). Experimental and theoretical investigation of anisotropic ion conduction of two-dimensional metal-organic frameworks. Inorg. Chem..

[cit40] Ohtani R., Xu J. E., Yanagisawa J., Iwai Y., Ehara T., Miyata K., Onda K., Pirillo J., Hijikata Y., Hiraoka T., Hayami S., Le Ouay B., Ohba M. (2023). Structural-transformation-induced Drastic Luminescence Changes in an Organic-Inorganic Hybrid [ReN(CN)] Salt Triggered by Chemical Stimuli. Angew. Chem., Int. Ed..

[cit41] Liberka M., Zakrzewski J. J., Heczko M., Reczynski M., Ohkoshi S., Chorazy S. (2021). Solvent- and Temperature-Driven Photoluminescence Modulation in Porous Hofmann-Type Sr-Re Metal-Organic Frameworks. Inorg. Chem..

[cit42] Seike M., Nagata K., Ikeda H., Ito A., Sakuda E., Kitamura N., Shinohara A., Yoshimura T. (2019). Synthesis and Photoluminescence of Tetracyanidonitridorhenium(v) Complexes with Five-Membered N-Heteroaromatic Ligands and Photoluminescence-Intensity Change. ACS Omega.

[cit43] Ikeda H., Ito A., Sakuda E., Kitamura N., Takayama T., Sekine T., Shinohara A., Yoshimura T. (2013). Excited-State Characteristics of Tetracyanidonitridorhenium(v) and -technetium(v) Complexes with N-Heteroaromatic Ligands. Inorg. Chem..

[cit44] Ikeda H., Yoshimura T., Ito A., Sakuda E., Kitamura N., Takayama T., Sekine T., Shinohara A. (2012). Photoluminescence Switching with Changes in the Coordination Number and Coordinating Volatile Organic Compounds in Tetracyanidonitridorhenium(v) and -technetium(v) Complexes. Inorg. Chem..

[cit45] Ohtani R., Yoshino H., Yanagisawa J., Ohtsu H., Hashizume D., Hijikata Y., Pirillo J., Sadakiyo M., Kato K., Shudo Y., Hayami S., Le Ouay B., Ohba M. (2021). Flexibility Control of Two-Dimensional Coordination Polymers by Crystal Morphology: Water Adsorption and Thermal Expansion. Chem.–Eur. J..

[cit46] Ohtani R., Grosjean A., Ishikawa R., Yamamoto R., Nakamura M., Clegg J. K., Hayami S. (2017). Zero in-Plane Thermal Expansion in Guest-Tunable 2D Coordination Polymers. Inorg. Chem..

[cit47] Nowick A. S., Vaysleyb A. V. (1997). Isotope effect and proton hopping in high-temperature protonic conductors. Solid State Ionics.

[cit48] Arcis H., Plumridge J., Tremaine P. R. (2022). Limiting Conductivities of Strong Acids and Bases in D2O and H2O: Deuterium Isotope Effects on Proton Hopping over a Wide Temperature Range. J. Phys. Chem. B.

[cit49] Su Y., Zheng J. J., Otake K., Hosono N., Kitagawa S., Gu C. (2024). Controlling Guest Diffusion by Local Dynamic Motion in Soft Porous Crystals to Separate Water Isotopologues and Similar Gases. Acc. Chem. Res..

[cit50] Lim D. W., Sadakiyo M., Kitagawa H. (2019). Proton transfer in hydrogen-bonded degenerate systems of water and ammonia in metal-organic frameworks. Chem. Sci..

[cit51] Zheng B., Wang L. L., Du L., Pan Y., Lai Z., Huang K. W., Du H. L. (2016). Diffusion as a function of guest molecule length and functionalization in flexible metal-organic frameworks. Mater. Horiz..

[cit52] Kreuer K. D., Rabenau A., Weppner W. (1982). Vehicle Mechanism, a New Model for the Interpretation of the Conductivity of Fast Proton Conductors. Angew. Chem. Int. Ed..

[cit53] Lu J. F., Yoshida Y., Maesato M., Kitagawa H. (2022). High-Performance All-Solid-State Proton Rectifier Using a Heterogeneous Membrane Composed of Coordination Polymer and Layered Double Hydroxide. Angew. Chem., Int. Ed..

[cit54] Lu J., Zhang H. C., Hou J., Li X. Y., Hu X. Y., Hu Y. X., Easton C. D., Li Q. Y., Sun C. H., Thornton A. W., Hill M. R., Zhang X. W., Jiang G. P., Liu J. Z., Hill A. J., Freeman B. D., Jiang L., Wang H. T. (2020). Efficient metal ion sieving in rectifying subnanochannels enabled by metal-organic frameworks. Nat. Mater..

[cit55] Liu Q., Xiao K., Wen L. P., Lu H., Liu Y. H., Kong X. Y., Xie G. H., Zhang Z., Bo Z. S., Jiang L. (2015). Engineered Ionic Gates for Ion Conduction Based on Sodium and Potassium Activated Nanochannels. J. Am. Chem. Soc..

[cit56] Tsuji Y., Ohtani R. (2025). Rectified water migration behavior in the noncentrosymmetric channels of a ferroelectric proton conductor. Inorg. Chem..

[cit57] Gao Q. L., Wang J. Q., Sanson A., Sun Q., Liang E. J., Xing X. R., Chen J. (2020). Discovering Large Isotropic Negative Thermal Expansion in Framework Compound AgB(CN) *via* the Concept of Average Atomic Volume. J. Am. Chem. Soc..

[cit58] Sapnik A. F., Geddes H. S., Reynolds E. M., Yeung H. H. M., Goodwin A. L. (2018). Compositional inhomogeneity and tuneable thermal expansion in mixed-metal ZIF-8 analogues. Chem. Commun..

[cit59] Liu Z. N., Gao Q. L., Chen J., Deng J. X., Lin K., Xing X. R. (2018). Negative thermal expansion in molecular materials. Chem. Commun..

[cit60] Hodgson S. A., Adamson J., Hunt S. J., Cliffe M. J., Cairns A. B., Thompson A. L., Tucker M. G., Funnell N. P., Goodwin A. L. (2014). Negative area compressibility in silver(i) tricyanomethanide. Chem. Commun..

[cit61] Lock N., Wu Y., Christensen M., Cameron L. J., Peterson V. K., Bridgeman A. J., Kepert C. J., Iversen B. B. (2010). Elucidating Negative Thermal Expansion in MOF-5. J. Phys. Chem. C.

[cit62] Wu Y., Kobayashi A., Halder G. J., Peterson V. K., Chapman K. W., Lock N., Southon P. D., Kepert C. J. (2008). Negative Thermal Expansion in the Metal-Organic Framework Material Cu(1,3,5-benzenetricarboxylate). Angew. Chem., Int. Ed..

[cit63] Lama P., Hazra A., Barbour L. J. (2019). Accordion and layer-sliding motion to produce anomalous thermal expansion behaviour in 2D-coordination polymers. Chem. Commun..

[cit64] Sergeenko A. S., Ovens J. S., Leznoff D. B. (2018). Designing anisotropic cyanometallate coordination polymers with unidirectional thermal expansion (TE): 2D zero and 1D colossal positive TE. Chem. Commun..

[cit65] Hibble S. J., Chippindale A. M., Pohl A. H., Hannon A. C. (2007). Surprises from a simple material–The structure and properties of nickel cyanide. Angew. Chem., Int. Ed..

[cit66] Iwai Y., Imamura Y., Nakaya M., Inada M., Le Ouay B., Ohba M., Ohtani R. (2023). Janus-Type Mixed-Valent Copper-Cyanido Honeycomb Layers. Inorg. Chem..

