# Supplementary material for: A ferroelectric proton conductor with colossal polarization induced by in-plane symmetry breaking in a two-dimensional coordination polymer
Source: Chem Sci. 2025 Jun 26;16(29):13413–21. doi: 10.1039/d4sc08700c (PMC12198974; doi:10.1039/d4sc08700c)
Supplement: SC-016-D4SC08700C-s001 [file SC-016-D4SC08700C-s001.pdf]

Supplementary Information (SI) for Chemical Science.
This journal is © The Royal Society of Chemistry 2025

Supporting information

Ferroelectric proton conductor with colossal polarization induced by in-plane symmetry breaking in a two-dimensional coordination polymer

Yanqing Song,[a] Yuta Tsuji,[b] Kunihisa Sugimoto,[c] Takashi Kikuchi,[d] Yuxin Shi,[a] Yusuke Murakami,[e] Kotaro Hiramatsu,[a] Benjamin Le Ouay,[a] Masaaki Ohba[a]* and Ryo Ohtani[a]*

[a.] Department of Chemistry, Faculty of Science, Kyushu University, 744 Motooka, Nishi-ku, Fukuoka 819-0395, Japan

[b.] Faculty of Engineering Sciences, Kyushu University, Kasuga, Fukuoka 816-8580, Japan

[c.] Department of Chemistry, Kindai University, 3-4-1 Kowakae, Higashi-osaka, Osaka 577-8502, Japan

[d.] Rigaku Corporation, 3-9-12 Matsubara-cho, Akishima-shi, Tokyo 196-8666, Japan

[e.] Ph.D. Program in Humanics, University of Tsukuba, 1-1-1 Tennodai, Tsukuba, Ibaraki 305-8577, Japan

Experimental sections

Synthesis: All chemicals were purchased from commercial sources and used without further purification. [Mn(salen)(H_2O)$_2$]Cl and (PPh_4)$_2$[ReN(CN)$_4$(MeOH)]·3MeOH were synthesized according to the methods described previously (ref43 in main manuscript).

[Mn(salen)]$_2$[ReN(CN)$_4$(MeCN)]·H_2O (MnReMeCN·H_2O): A solution of (PPh_4)$_2$[ReN(CN)$_4$]·3MeOH (215.6 mg, 4 mmol) in MeCN (100 mL) was added to a solution of [Mn(salen)(H_2O)$_2$]Cl (158.1 mg, 2 mmol) in H_2O (100 mL) at room temperature. Brown block shaped single crystals of **MnReMeCN·H_2O** were obtained after the mixture was left for 3 days. Powder samples of **MnReMeCN·H_2O** were prepared by grinding the corresponding single crystals. Anal.Found (calcd) for $C_{38}H_{33}Mn_2N_{10}O_5Re$: C 45.05 (45.38), H 3.24 (3.31), N 13.75 (13.93).

Physical Measurement: Diffraction data for **MnReMeCN·H_2O** were collected at 100 K using a PILATUS3 X CdTe 1M detector (DECTRIS) at the SPring-8/BL02B1 beamline in Japan. The wavelengths (λ = 0.4139 Å) were selected using Si(311) monochromator crystals. Data were collected and processed using the RAPID-AUTO program (Rigaku) and were corrected for Lorentz and polarization effects. Diffraction data for **MeReMeCN** were collected at 150, 170, 200, 250, 270, 300, 330, 350, 400, and 420 K using an XtaLAB Synergy R, DW system with MoKα radiation (λ = 0.71075 Å) from a PhotonJet-R rotating anode X-ray source equipped with multilayer mirror optics. Each single crystal was mounted on a MicroMount using paraffin oil. The temperature of sample was controlled by a dry dinitrogen flow using an Oxford cryosystems plus. Diffraction Data integration and reduction were undertaken with CrysAlisPro. Using Olex2 crystallography software, the structures were solved with the intrinsic phasing method by using the SHELXT [ref. 1]. Structural refinements were conducted by the full-matrix least-square method using SHELXL [ref. 2]. None-hydrogen atoms were refined anisotropically, and all hydrogen atoms were refined using the riding model.

Powder X-ray diffraction (PXRD) data were collected with Miniflex 600-C (Rigaku) with a D/teX Ultra II detector using CuKα radiation (λ = 1.54184 Å) and a reflection-free sample holder in ambient air at room temperature. Thermogravimetric analysis (TGA) was performed using a Rigaku Instrument Thermo plus TG 8120 in the range of 303-773 K, under N_2 atmosphere (heating rate: 10 K min^{-1}). The C, H and N elemental analyses for samples were carried out at the division of graduate school of science in Kyushu University.

AC impedance measurements were conducted using an SP-300 (Biologic) over a frequency range of 7 MHz to 0.1 Hz, with an input voltage amplitude of 50 mV. Humidity was controlled using a constant-temperature and humidity chamber SH-242 (Espec). Measurements were performed after a stabilization period of at least 2 h, once the target humidity and temperature were reached. Gold wires

were attached to both ends of the [100] and [0-11] ([0-1-1]) directions of each crystal, with silver paste used as the blocking electrode.

Ferroelectricity measurements were performed using an axiACT TF Analyzer 1000 by Dynamic Hysteresis Mode (single-pulse mode) and Positive-Up-Negative-Down (PUND) methods.

The water-adsorption isotherm was measured with a BELSORP-MAX volumetric adsorption instrument. The samples were dehydrated by heating at 400 K for 16 h before the measurements.

The magnetic properties were investigated using a Quantum Design MPMS-XL5R SQUID under an applied DC magnetic field of 1000 Oe.

Second harmonic generation (SHG) measurements were performed using a custom-built SHG microscope. The excitation light source was derived from a Yb-doped femtosecond oscillator (FLINT FL2, Light Conversion), whose pulses were spectrally broadened using a photonic crystal fiber (Thorlabs, LMA-PM-5). The broadened pulses were then compressed using a combination of chirped mirrors and a prism compressor to achieve near-transform-limit pulses. For sample excitation, an oil immersion objective lens (OLYMPUS, UPlanXApo 60x NA1.42) was used to tightly focus the laser beam. The generated SHG signal was collected with another objective lens (Nikon, CFI Plan Apo 20x NA 0.75) and then analyzed using a custom-built spectrometer equipped with a CCD (Princeton Instruments, PIXIS 100 BR) after eliminating the incident laser using a short-pass filter (Thorlabs, FESH0750). The SHG spectrum (Fig. S5a and S5f) was acquired with an excitation power of 15 mW before the microscope and an acquisition time of 50 ms. In the excitation power dependence study (Fig. S5b), SHG intensities at 509 nm was plotted as a function of the excitation power. In the time-dependent SHG meausurements (Fig. S5c), SHG intensities at a single position was measured over 600 s, where the excitation light was continuously irradiated to the sample to induce thermal dehydration of the sample. The SHG images (Fig. S5d) were acquired by taking a series of SHG spectra at different positions by moving the sample with a DC linear motor stage (MLS203, Thorlabs).

DFT calculation: The crystal structure of **MnReMeCN·H_2O** was optimized using the Vienna Ab initio Simulation Package (VASP) [ref. 3, 4] until the forces on all atoms were reduced to less than 0.02 eV/Å. During this process, the shape of the unit cell was maintained while optimizing the cell volume and allowing the ionic degrees of freedom to relax. The generalized gradient approximation was used, as described by Perdew, Burke, and Ernzerhof [ref. 5]. The Kohn–Sham equations were solved using a plane-wave basis set with the projector-augmented wave method [ref. 6], with a cutoff energy of 500 eV for the plane-wave basis set. The convergence threshold for self-consistent field iteration was set to 1.0×10^{-6} eV. Only Γ-point was used to sample the Brillouin zone. Grimme's D3 dispersion correction with Becke–Johnson damping [ref. 7] was applied. The optimized crystal structure obtained in this manner is shown in Figure S3a. The optimized structure is visualized here using VESTA [ref. 8].

Polarization calculation: The spontaneous polarization of **MnReMeCN·H_2O** was calculated using the Berry phase method implemented in VASP [ref. 9]. To create the reference non-polar structure, the Re≡N bonds were rotated around the b-axis, with each Re atom serving as a center, aligning the Re≡N bonds parallel to the a-axis. During this process, the acetonitrile and CN ligands coordinated to each Re atom were also rotated to maintain their relative positions. The structure created in this manner is shown in Figure S3b. The spontaneous polarization was obtained by monitoring the change in polarization of an arbitrary chosen branch of the polarization lattice shown in Figure S3c, when the structure is deformed from the non-polar structure (Figure S3b) to the polar structure (Figure S3a). The intermediate structures were generated through linear interpolation.

Water migration calculation: A potential energy surface (PES) scan was performed for the migration of the four water molecules in the a-, b-, and c-axis directions. For the a- and b-axes, only one calculation was performed for each axis, focusing on water migration in the positive direction. In contrast, for the c-axis, two calculations were conducted: one for water migration in the positive direction and the other in the negative direction. The water molecules were displaced incrementally by 0.1 Å along each axis direction, and at each step, the atomic coordinates within the unit cell were relaxed. However, the coordinates along the displacement axis for the oxygen atoms of the water molecules and the Mn atoms in the framework remained fixed. To reduce computational costs, the force convergence criterion during optimization at each step was set to a slightly relaxed value of 0.03 eV/Å, while all other calculation conditions remained the same as described above. Since the unit cell contains four water molecules, the energy change calculated in this manner corresponds to the migration of four water molecules. Therefore, by dividing it by four, the energy change per water molecule was obtained and used to plot the PES.

1) Sheldrick GM. SHELXT - Integrated space-group and crystal-structure determination. *Acta Crystallogr A.* **2015**, *71*, 3-8, DOI:10.1107/S2053273314026370

2) Sheldrick GM. Crystal structure refinement with SHELXL. *Acta Crystallogr C Struct Chem.* **2015**, *71*, 3-8, DOI:10.1107/S2053229614024218

3) Kresse, G.; Furthmüller, J. Efficiency of Ab-Initio Total Energy Calculations for Metals and Semiconductors Using a Plane-Wave Basis Set. *Comput. Mater. Sci.* **1996**, *6*, 15– 50, DOI: 10.1016/0927-0256(96)00008-0

4 Kresse, G.; Furthmüller, J. Efficient Iterative Schemes for Ab Initio Total-Energy Calculations Using a Plane-Wave Basis Set. *Phys. Rev. B: Condens. Matter Mater. Phys.* **1996**, *54*, 11169–

11186, DOI: 10.1103/PhysRevB.54.11169

5) Perdew, J. P.; Burke, K.; Ernzerhof, M. Generalized Gradient Approximation Made Simple. *Phys. Rev. Lett.* **1996**, *77*, 3865– 3868, DOI: 10.1103/PhysRevLett.77.3865

6) Kresse, G.; Joubert, D. From Ultrasoft Pseudopotentials to the Projector Augmented-Wave Method. *Phys. Rev. B: Condens. Matter Mater. Phys.* **1999**, *59*, 1758– 1775, DOI: 10.1103/PhysRevB.59.1758

7) Grimme, S.; Ehrlich, S.; Goerigk, L. Effect of the Damping Function in Dispersion Corrected Density Functional Theory. *J. Comput. Chem.* **2011**, *32*, 1456– 1465, DOI: 10.1002/jcc.21759

8) Momma, K.; Izumi, F. VESTA 3 for Three-Dimensional Visualization of Crystal, Volumetric and Morphology Data. *J. Appl. Crystallogr.* **2011**, *44*, 1272– 1276, DOI: 10.1107/S0021889811038970

9) King-Smith, R. D.; Vanderbilt, D. Theory of Polarization of Crystalline Solids. *Phys. Rev. B: Condens. Matter Mater. Phys.* **1993**, *47*, 1651– 1654, DOI: 10.1103/physrevb.47.1651

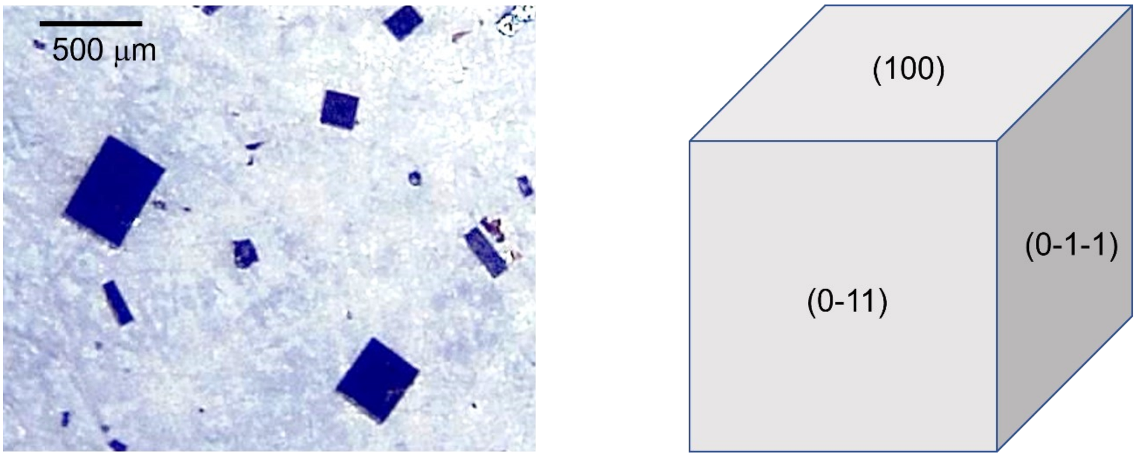

Figure S1 Picture of single crystals of **MnReMeCN·H_2O** and its face index result.

Table S1 Crystal parameters of **MnReMeCN·H_2O**

**Temp. (K)**	**100**
**CCDC number**	2410485
**Crystal System**	Orthorhombic
**Space Group**	$Pna2_1$
**Formula**	$C_{38}H_{26}Mn_2N_{10}O_5Re_1$
a **(Å)**	17.9573(3)
b **(Å)**	14.2990(2)
c **(Å)**	14.6854(2)
V **(Å^3)**	3770.79(10)
**Z**	4
**R1**	4.22
**Rwp**	12.47
**G.O.F**	1.071
**Flack Parameter**	0.394(5)

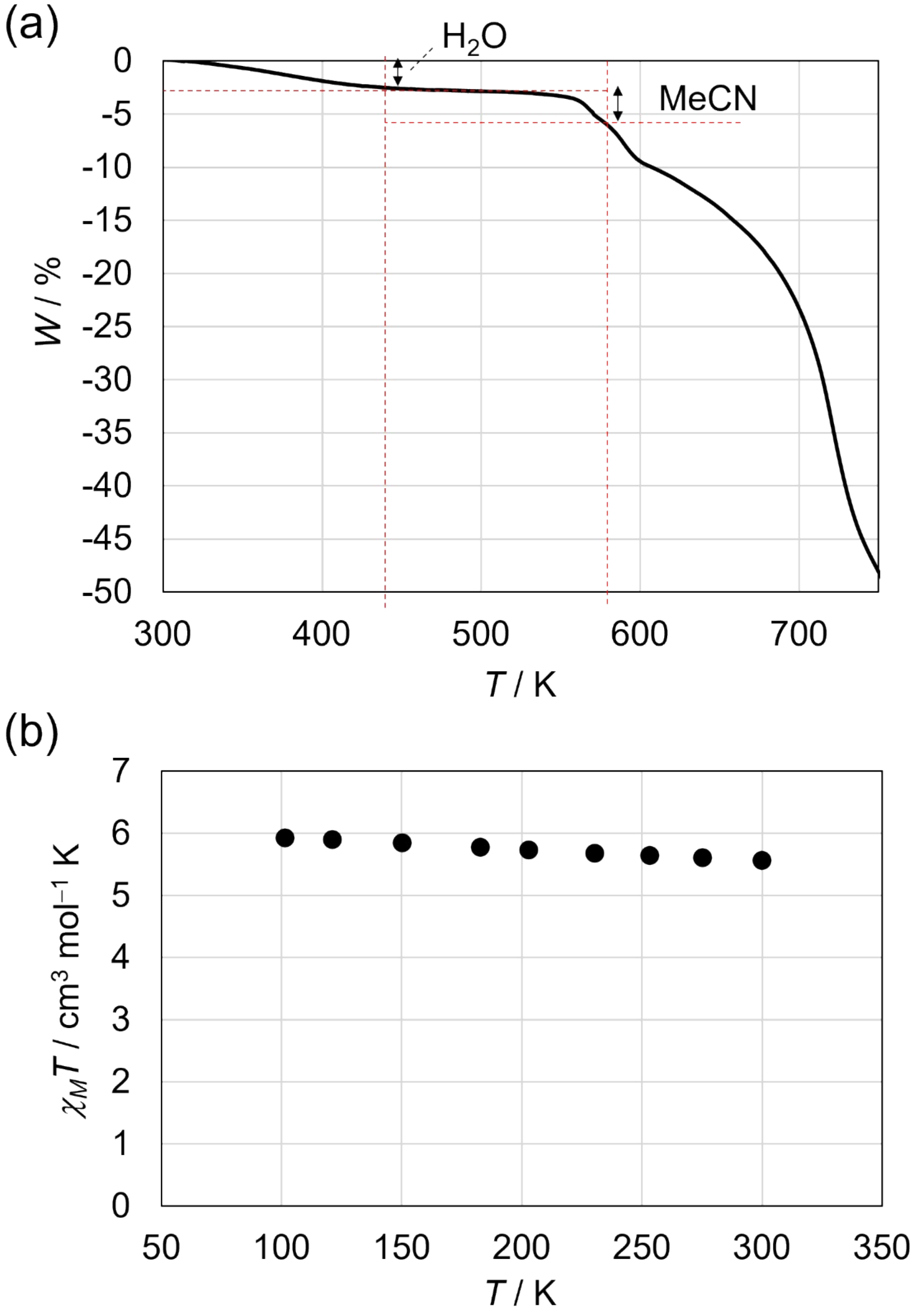

Figure S2 (a) TG curve of **MnReMeCN·H$_2$O**. (b) SQUID result for **MnReMeCN·H$_2$O**, demonstrating two Mn(III) (d^4; S = 2) and one Re(V) (d^2; S = 0) ions are incorporated.

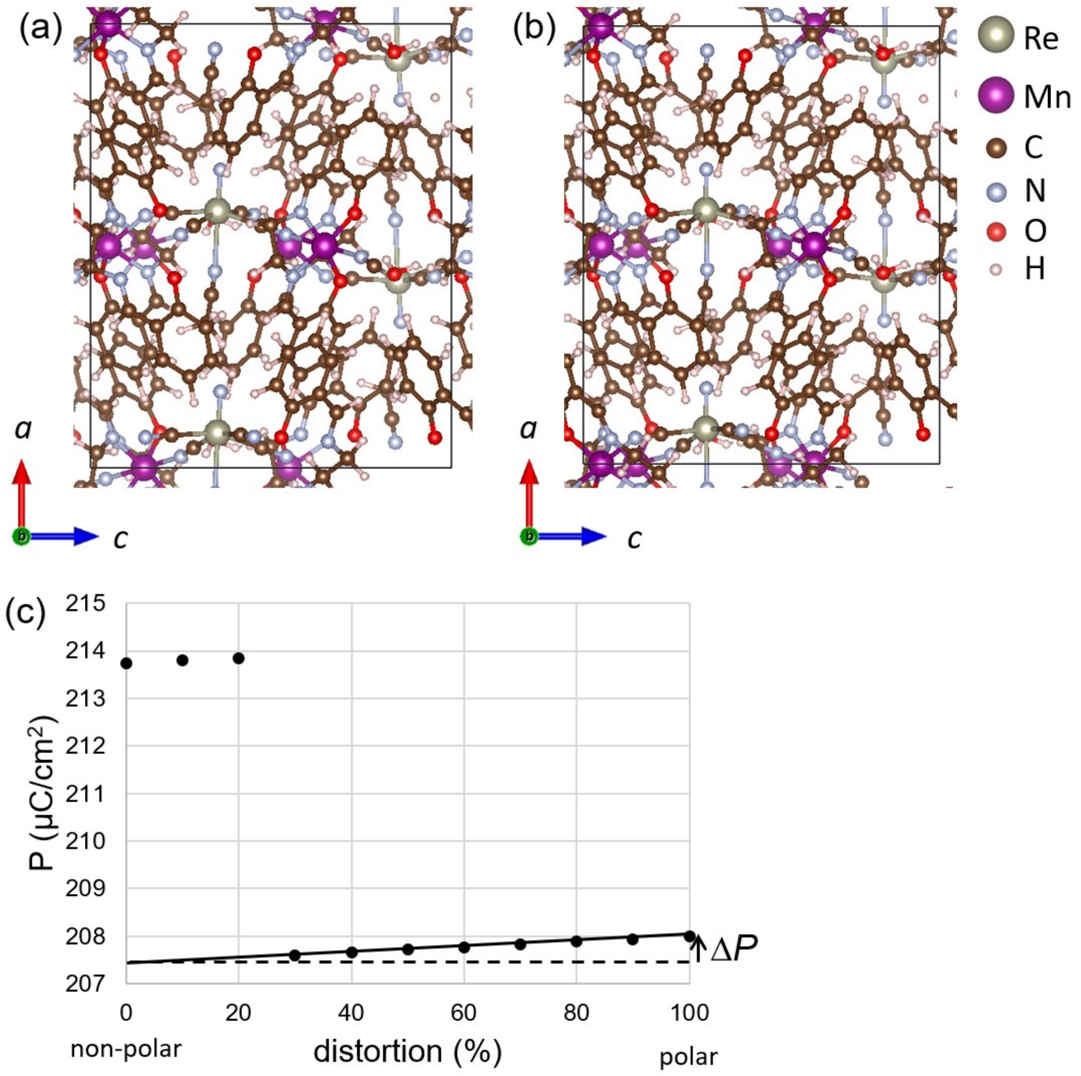

Figure S3. (a) Optimized crystal structure of **MnReMeCN·H_2O** with polarization. (b) The non-polar reference structure created based on the structure in (a). (c) Polarization lattice as a function of a distortion between the polar and non-polar structures. The black solid line represents the branch selected to calculate the polarization variation, resulting in ΔP = 0.57 μC/cm^2.

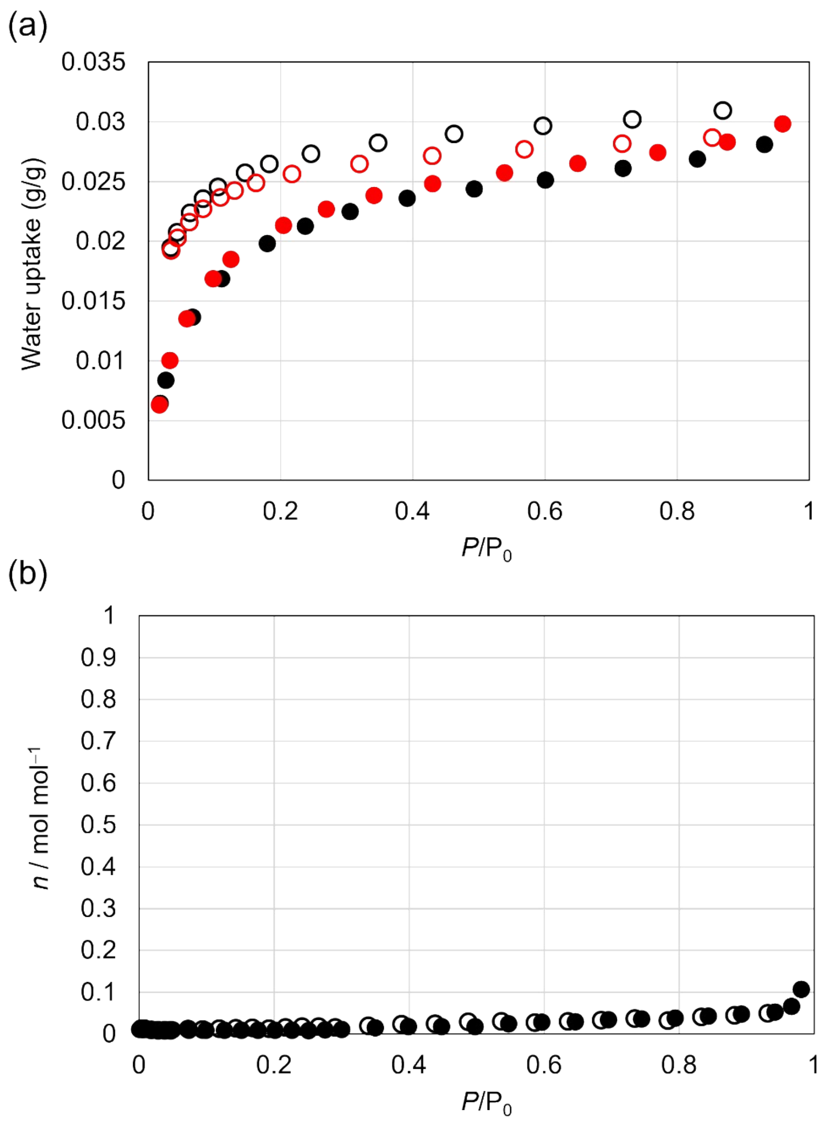

Figure S4 (a) Water adsorption isotherms of **MnReMeCN** at room temperature. The first and second cycles are shown by black and red circles, respectively. Close (adsorption), open (desorption). (b) N_2 adsorption isotherms of **MnReMeCN** at 77 K.

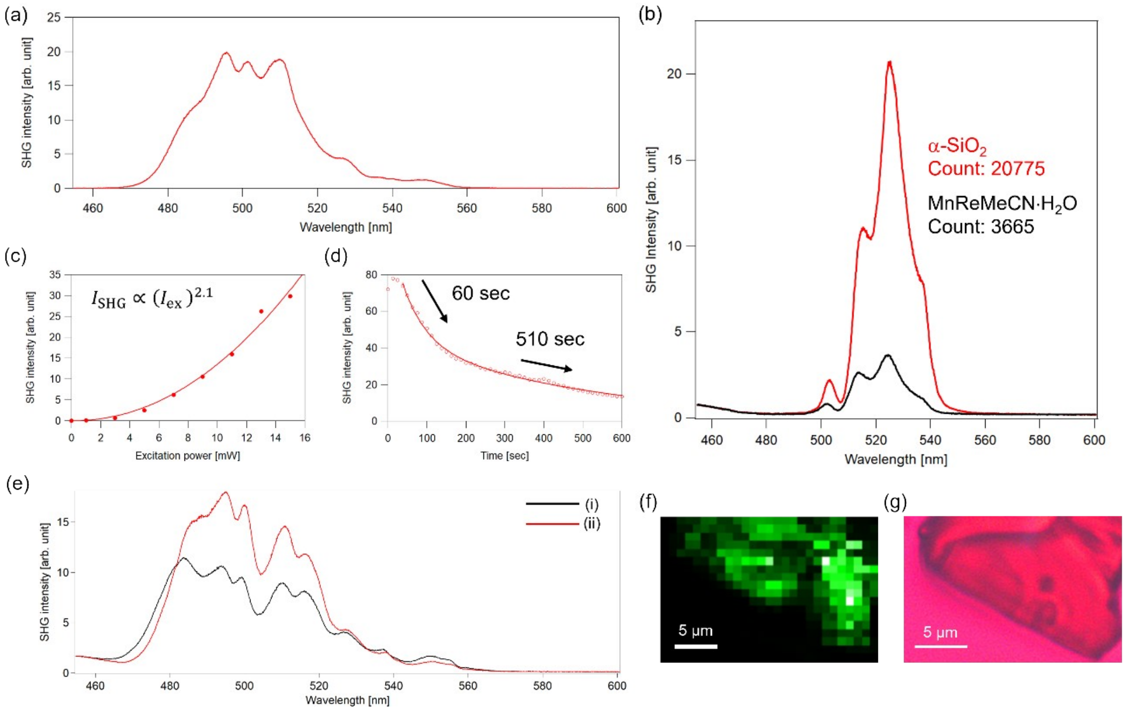

Figure S5 (a) The SHG spectrum of a selected crystal. (b) Comparison of the SHG intensities between MnReMeCN·H_2O and α-SiO_2. (c) The excitation power dependency of the SHG intensities at 509 nm. (d) The time-dependent SHG measurements. SHG intensities at a single position was measured over 600 s, where the excitation light was continuously irradiated to the sample to induce thermal dehydration of the sample. (e) The SHG spectra of (i) another crystal and (ii) a selected crystal after five-cycle of dehydration/rehydration. (f) The SHG images were acquired by taking a series of SHG spectra at different positions by moving the sample with a DC linear motor stage (MLS203, Thorlabs). (g) A real image of the corresponding crystal of (f).

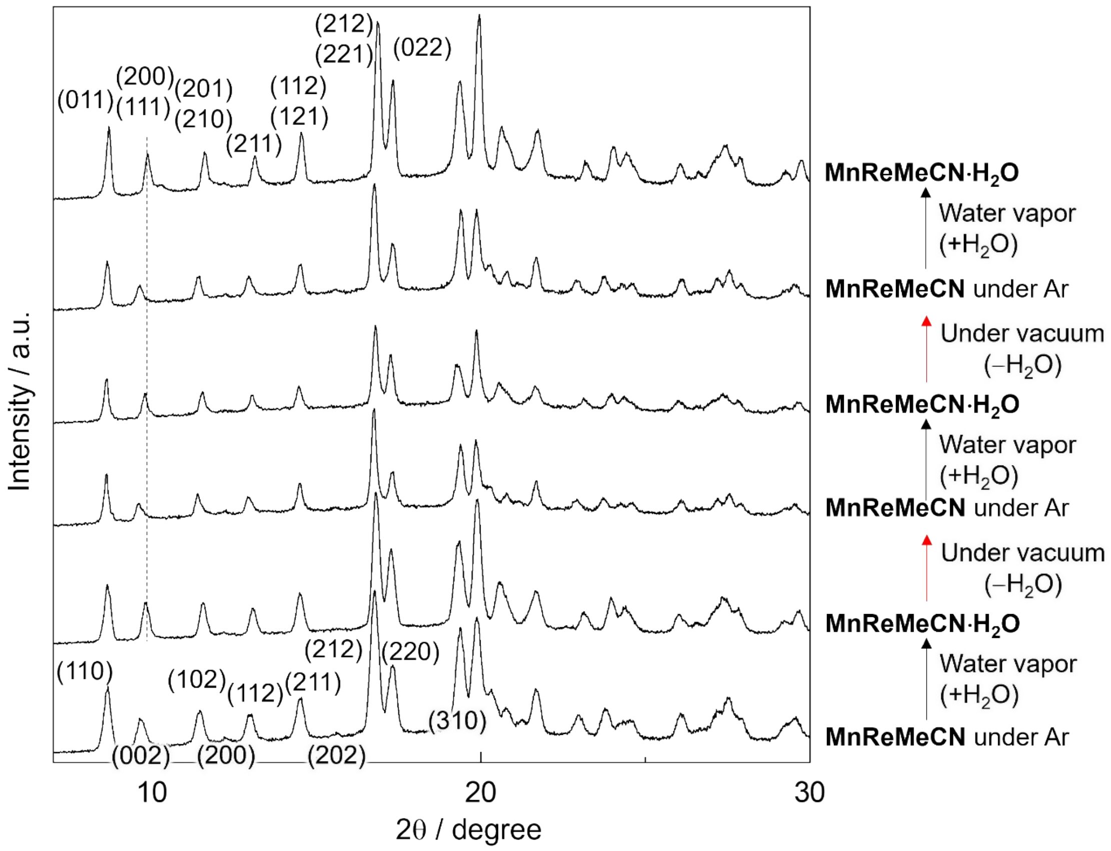

Figure S6 PXRD results demonstrating reversible structural transformation via water vapor between **MnReMeCN·H_2O** and **MnReMeCN**

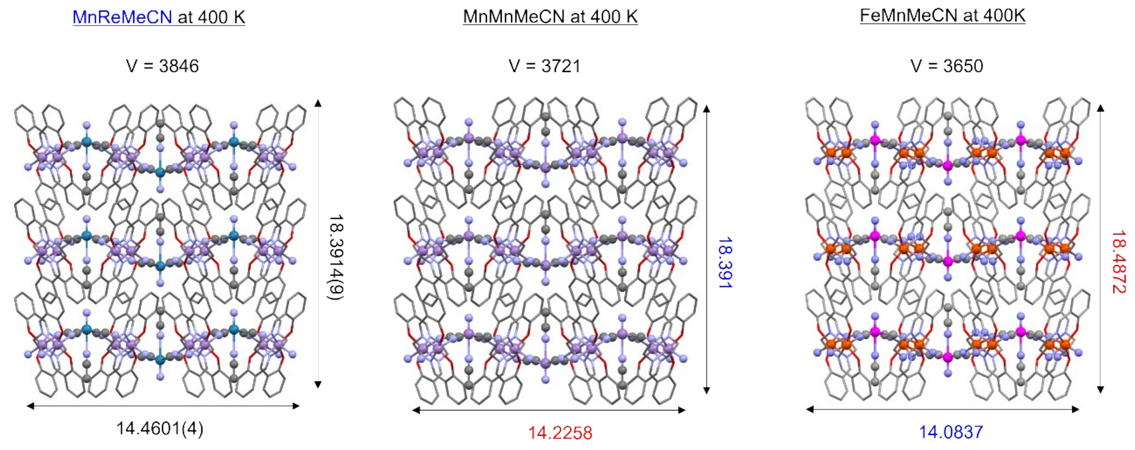

Figure S7 Comparison of crystal structures of three analogs at 400 K.

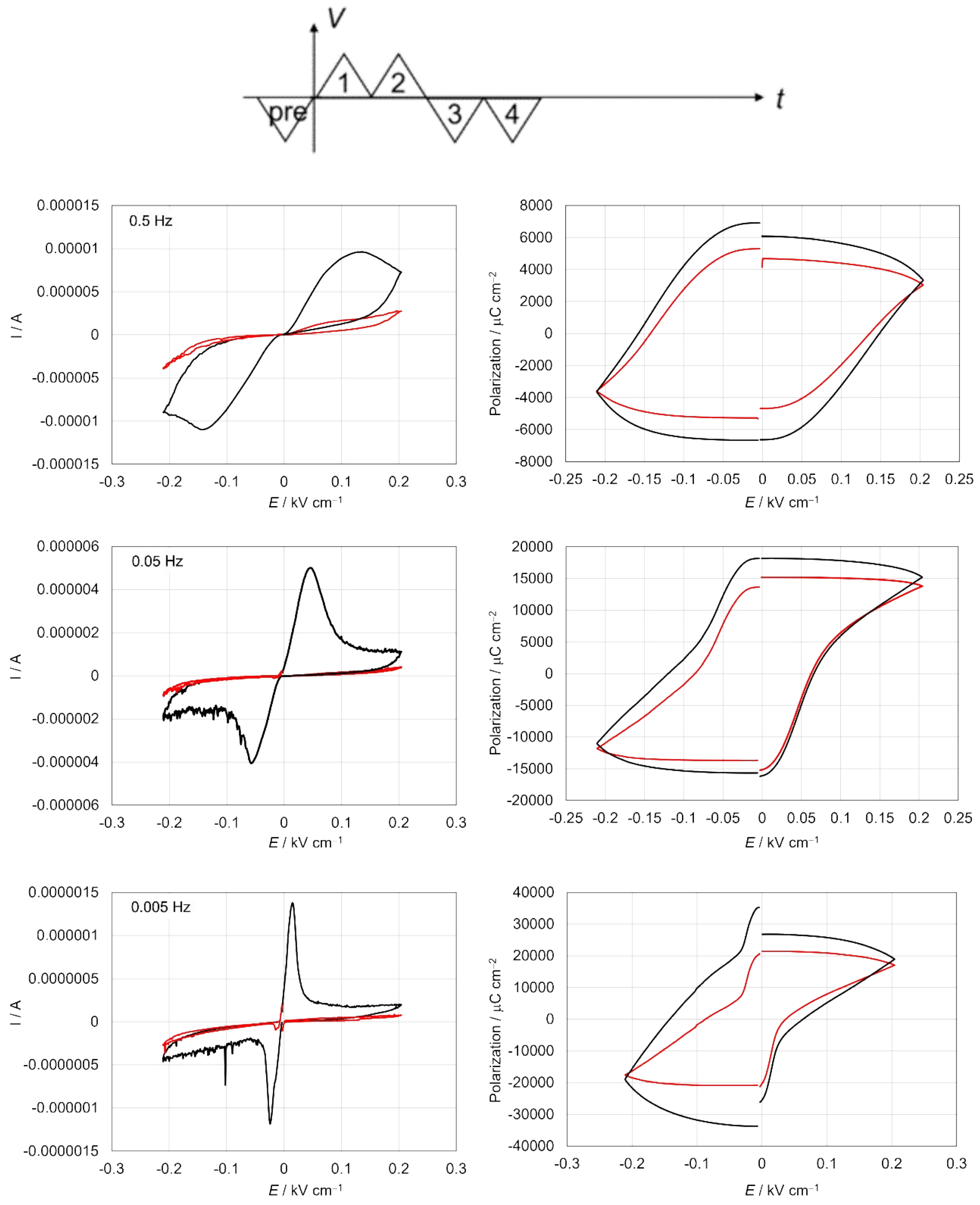

Figure S8 *I-E* curves (left; black lines are the first and third pulses; red lines are the second and fourth pulses) and hysteresis loops (right; black lines are loops drown by using currents of the first and third pulses; red lines are the loops obtained by using I_{first}–I_{second} and I_{third}–I_{fourth}) of the single crystal of **MnReMeCN·H_2O** obtained by the PUND methods at 0.5, 0.05, 0.005 Hz.

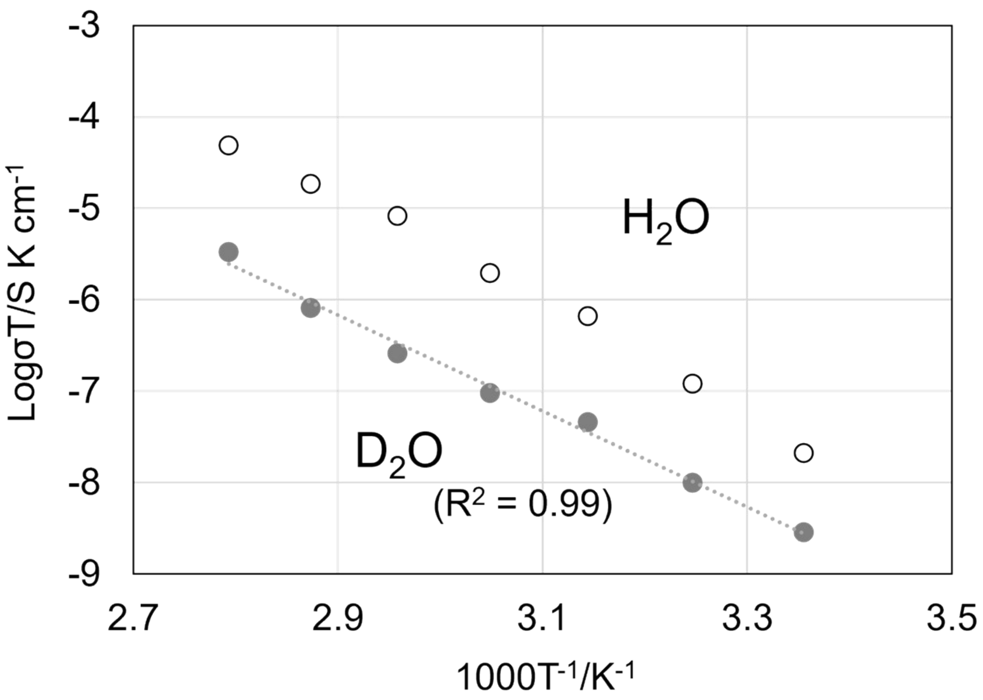

Figure S9 Arrhenius plots of **MnReMeCN·H_2O** and **MnReMeCN·D_2O**.

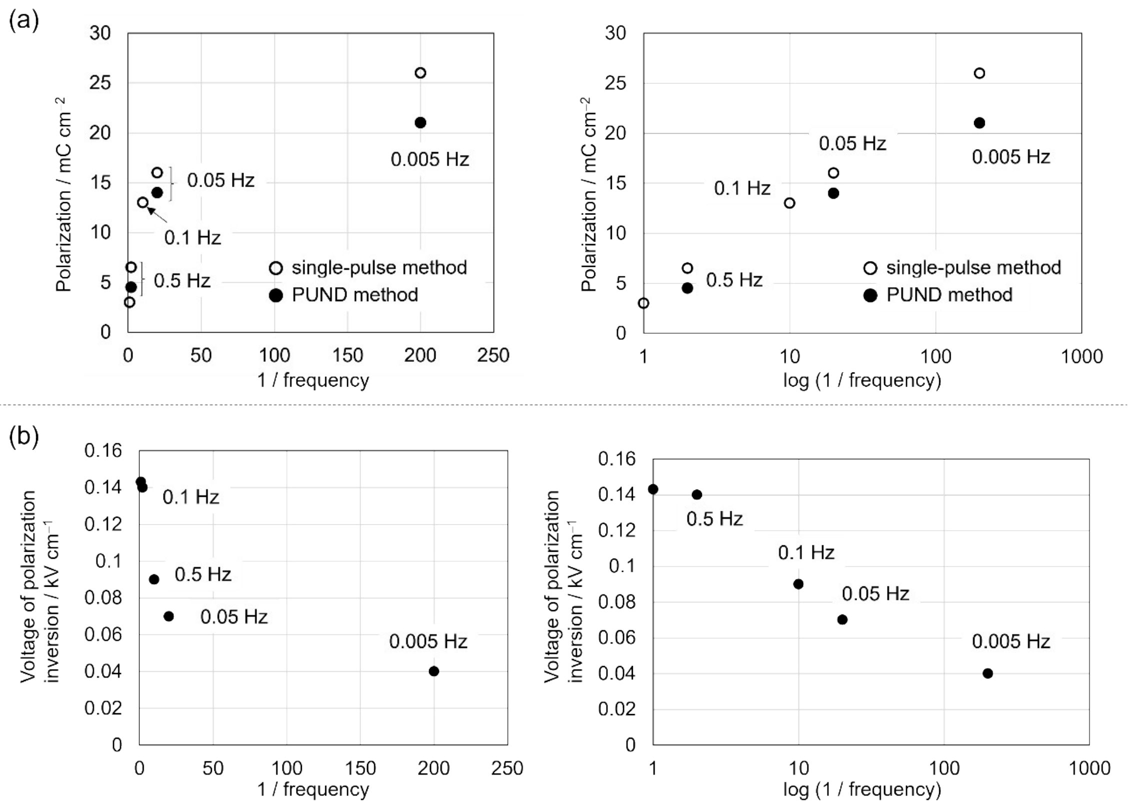

Figure S10 (a) Polarization as a function of frequency. (b) Voltage of polarization inversion as a function of frequency.

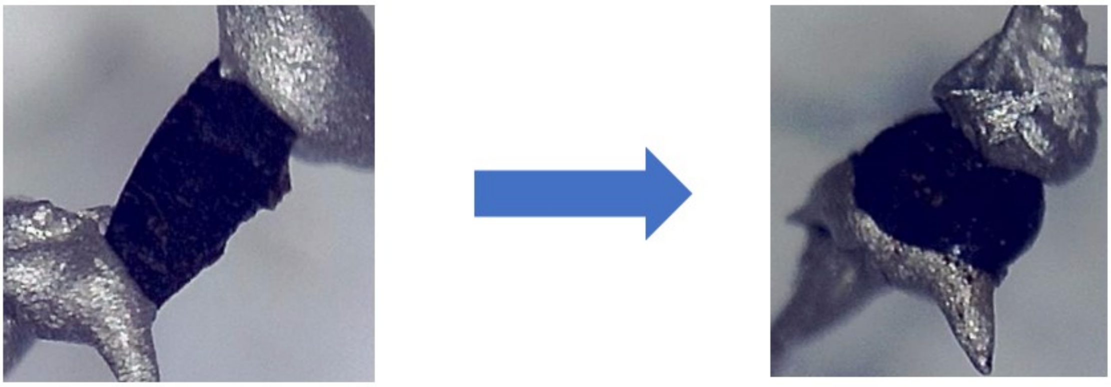

Figure S11 Pictures of the single crystal before and after a PE measurement with 100 V.

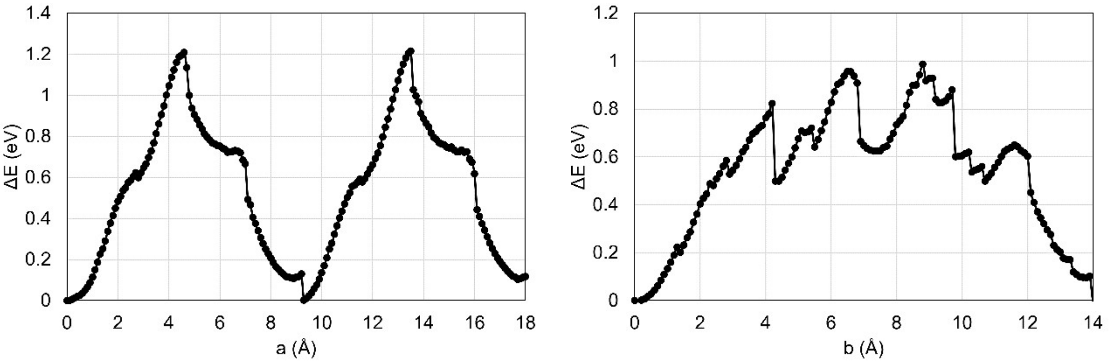

Figure S12 Changes in potential energy calculated for **MnReMeCN·H_2O** when the positions of the water molecules are displaced in the *a*- (left) and *b*- (right) axes.

(a)

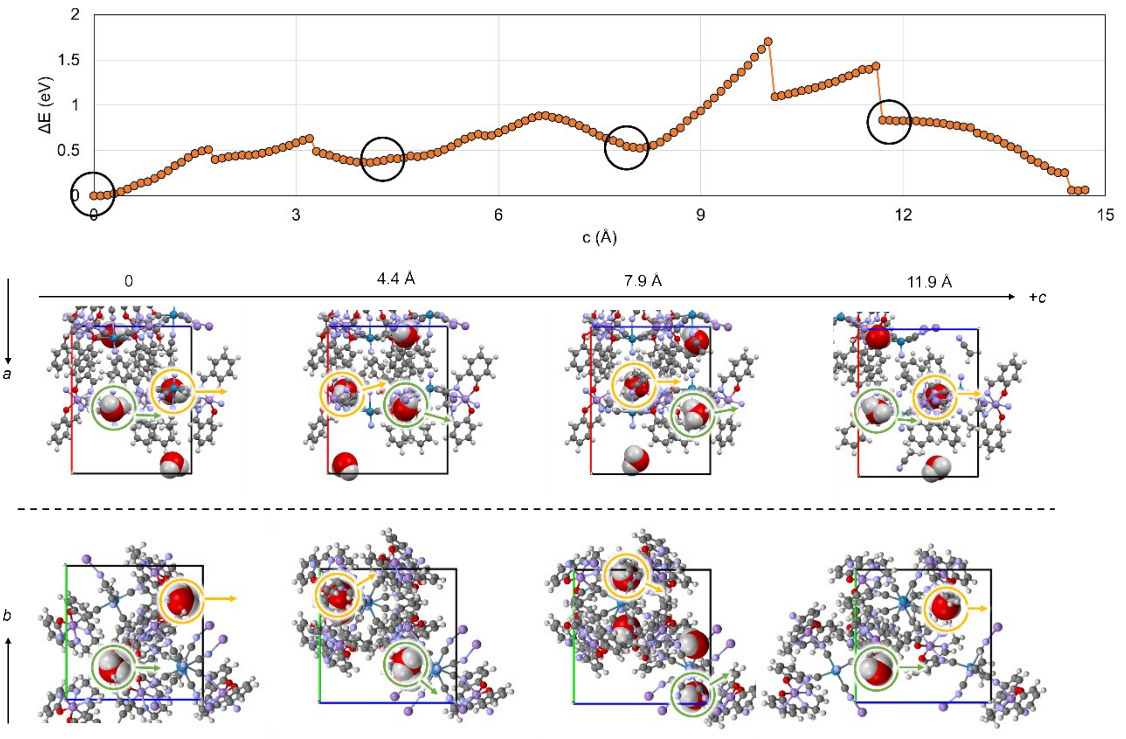

(b)

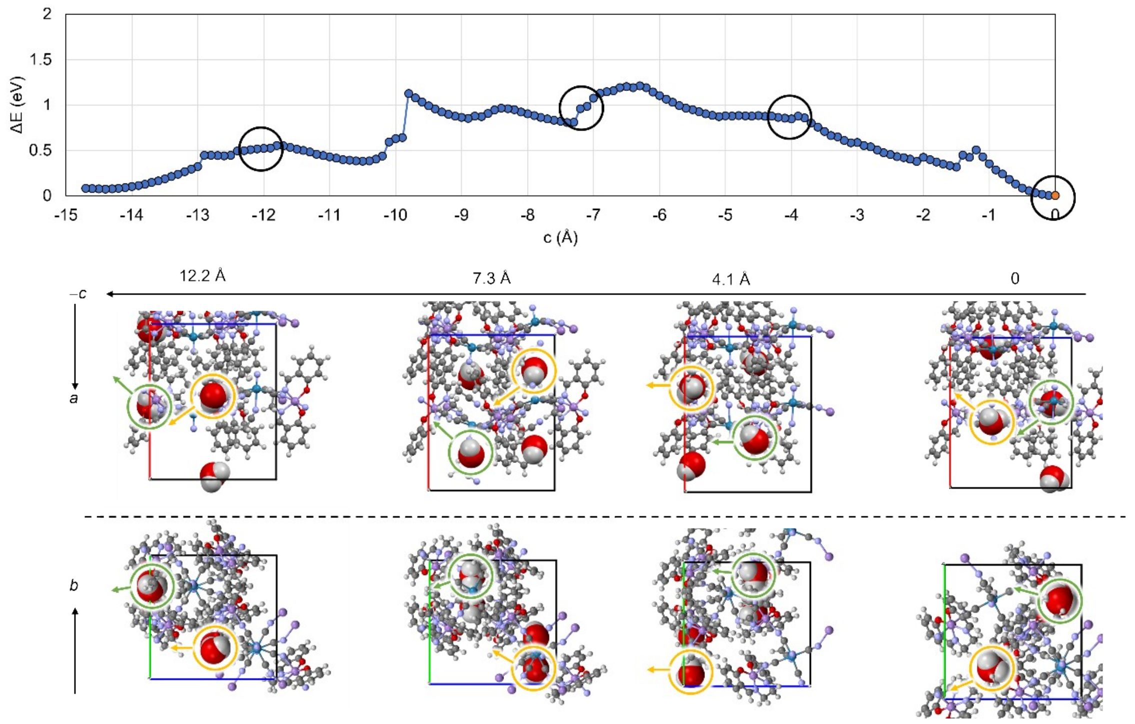

Figure S13 Overviews of the structures corresponding to the selected points in the (a) positive and (b) negative directions, where water molecules are highlighted.

Table S2. Crystal parameters of **MnReMeCN**

Temp. (K)	150	170	200
CCDC number	2410486	2410487	2410488
Crystal System	Orthorhombic	Orthorhombic	Orthorhombic
Space Group	*P*4/*n*	*P*4/*n*	*P*4/*n*
Formula	$C_{38}H_{31}Mn_2N_{10}O_4Re_1$	$C_{38}H_{31}Mn_2N_{10}O_4Re_1$	$C_{38}H_{31}Mn_2N_{10}O_4Re_1$
a (Å)	14.3719(3)	14.3742(3)	14.3878(3)
b (Å)	14.3719(3)	14.3742(3)	14.3878(3)
c (Å)	18.0827(6)	18.1157(6)	18.1690(6)
V (Å^3)	3735.0(2)	3743.0(2)	3761.1(2)
Z	4	4	4
R1	5.90	5.74	5.40
Rwp	14.82	14.46	14.06
G.O.F	1.131	1.138	1.124

Temp. (K)	250	270	300
CCDC number	2410489	2410490	2410491
Crystal System	Orthorhombic	Orthorhombic	Tetragonal
Space Group	*P*4/*n*	*P*4/*n*	*P*4/*ncc*
Formula	$C_{38}H_{31}Mn_2N_{10}O_4Re_1$	$C_{38}H_{31}Mn_2N_{10}O_4Re_1$	$C_{38}H_{31}Mn_2N_{10}O_4Re_1$
a (Å)	14.3963(3)	14.3992(3)	14.4061(3)
b (Å)	14.3963(3)	14.3992(3)	14.4061(3)
c (Å)	18.2533(7)	18.2847(7)	18.3403(8)
V (Å^3)	3783.1(2)	3791.1(2)	3806.3(2)
Z	4	4	4
R1	4.50	4.22	5.55
Rwp	10.69	9.67	10.22
G.O.F	1.178	1.183	1.347

**Temp. (K)**	**330**	**350**	**400**
**CCDC number**	2410492	2410493	2410494
**Crystal System**	Tetragonal	Tetragonal	Tetragonal
**Space Group**	*P*4/*ncc*	*P*4/*ncc*	*P*4/*ncc*
**Formula**	$C_{38}H_{31}Mn_2N_{10}O_4Re_1$	$C_{38}H_{31}Mn_2N_{10}O_4Re_1$	$C_{38}H_{31}Mn_2N_{10}O_4Re_1$
***a* (Å)**	14.4217(4)	14.4280(3)	14.4601(4)
***b* (Å)**	14.4217(4)	14.4280(3)	14.4601(4)
***c* (Å)**	18.3812(9)	18.3832(9)	18.3914(9)
***V* (Å^3)**	3823.0(3)	3826.8(2)	3845.5(3)
**Z**	4	4	4
**R1**	3.79	3.68	3.92
**Rwp**	8.21	8.24	8.39
**G.O.F**	1.122	1.093	1.116

**Temp. (K)**	**420**
**CCDC number**	2410495
**Crystal System**	Tetragonal
**Space Group**	*P*4/*ncc*
**Formula**	$C_{38}H_{31}Mn_2N_{10}O_4Re_1$
***a* (Å)**	14.4843(2)
***b* (Å)**	14.4843(2)
***c* (Å)**	18.3887(9)
***V* (Å^3)**	3857.9(3)
**Z**	4
**R1**	4.20
**Rwp**	9.38
**G.O.F**	1.137

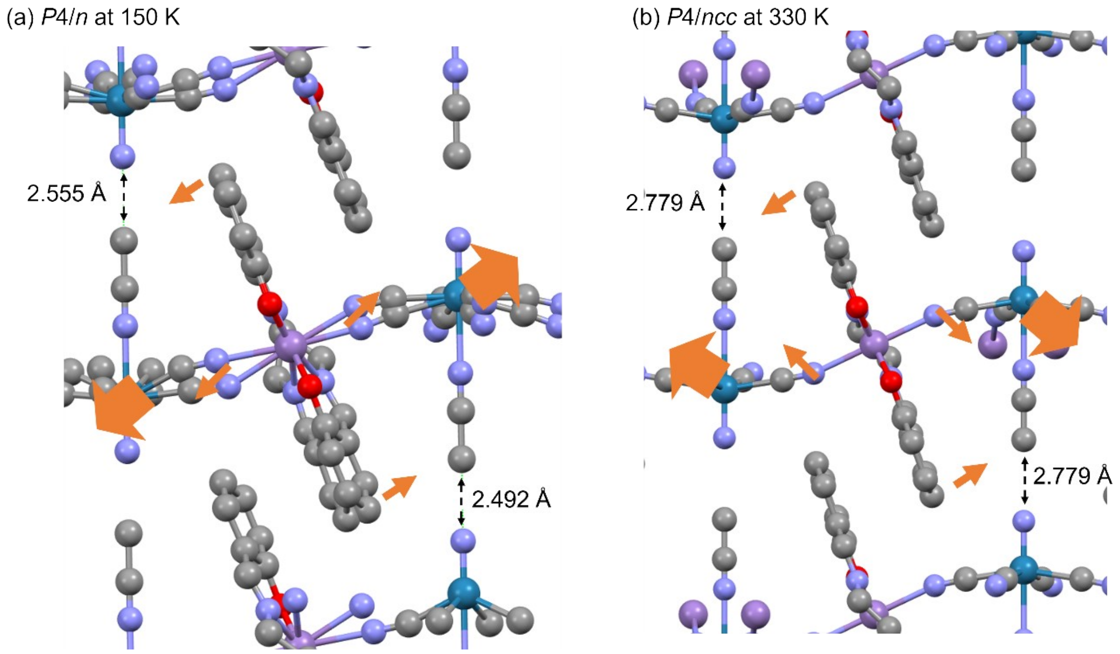

Figure S14 Schematics of structural transformation of layers during thermal expansion of (a) **MnReMeCN$_{\mathbf{LT}}$** and (b) **MnReMeCN$_{\mathbf{HT}}$**.
